# Supplementary material for: Continuous Movement Monitoring at Home Through Wearable Devices: A Systematic Review
Source: Sensors (Basel). 2025 Aug 8;25(16):4889. doi: 10.3390/s25164889 (PMC12389529; doi:10.3390/s25164889)
Supplement: Supplementary file 1 [file sensors-25-04889-s001.zip › Appendix S1.pdf]

Appendix S1. Search Strings

Pubmed: ("wearable" OR "inertial" OR "remote" OR "continuous" OR "markerless") AND ("sensor*" OR "assessment" OR "monitoring") AND ("home") AND ("motor" OR "movement" OR "gait" OR "walk" OR "lower limb" OR "upper limb" OR "arm" OR "fall*").

Scopus: TITLE-ABS-KEY (("wearable" OR "inertial" OR "remote" OR "continuous" OR "markerless") AND ("sensor*" OR "assessment" OR "monitoring") AND ("home") AND ("motor" OR "movement" OR "gait" OR "walk" OR "lower limb" OR "upper limb" OR "arm" OR "fall*")).

Web of Science: TS = ("wearable" OR "inertial" OR "remote" OR "continuous" OR "markerless") AND TS = ("sensor*" OR "assessment" OR "monitoring") AND TS = ("home") AND TS = ("motor" OR "movement" OR "gait" OR "walk" OR "lower limb" OR "upper limb" OR "arm" OR "fall.
