# Supplementary material for: Continuous Movement Monitoring at Home Through Wearable Devices: A Systematic Review
Source: Sensors (Basel). 2025 Aug 8;25(16):4889. doi: 10.3390/s25164889 (PMC12389529; doi:10.3390/s25164889)
Supplement: Supplementary file 1 [file sensors-25-04889-s001.zip › Appendix S2.pdf]

Appendix S2. Data extraction

Part 1 of 3: Data extraction on Title, Authors, DOI, Year and Country of the included publications

Title	Authors	DOI	Year	Country	Reference number
Detection of Wandering Behaviors Using a Body-Worn Inertial Sensor in Patients With Cognitive Impairment: A Feasibility Study	Kamil, RJ and Bakar, D and Ehrenburg, M and Wei, EX and Pletnikova, A and Xiao, GC and Oh, ES and Mancini, M and Agrawal, Y	10.3389/fneur.2021.529661	2021	USA	[102]
Gait in Mild Alzheimer's Disease: Feasibility of Multi-Center Measurement in the Clinic and Home with Body-Worn Sensors: A Pilot Study	Mc Ardle, R and Morris, R and Hickey, A and Del Din, S and Koychev, I and Gunn, RN and Lawson, J and Zamboni, G and Ridha, B and Sahakian, et. Al and Deep & Frequent Phenotyping Study	10.3233/JAD-171116	2018	United Kingdom	[94]
Locomotor activities of individuals with lower-limb amputation	Srisuwan, B. and Klute, G.K.	10.1097/PXR.0000000000000009	2021	USA	[84]
A novel portable sensor to monitor bodily positions and activities in transtibial prosthesis users	Mertens, J.C. and Brzostowski, J.T. and Vamos, A. and Allyn, K.J. and Hafner, B.J. and Friedly, J.L. and DeGrasse, N.S. and Ballesteros, D. and Krout, A. and Larsen, et al	10.1016/j.clinbiomech.2022.105741	2022	USA	[33]
Wearable Sensors Quantify Mobility in People With Lower Limb Amputation During Daily Life	Kim, J and Colabianchi, N and Wensman, J and Gates, DH	10.1109/TNSRE.2020.2990824	2020	USA	[88]
Daily step count of British military males with bilateral lower limb amputations: A comparison of in-patient rehabilitation with the consecutive leave period between admissions	Sherman, K. and Roberts, A. and Murray, K. and Deans, S. and Jarvis, H.	10.1177/0309364618806058	2019	United Kingdom	[87]

Monitoring daily function in persons with transfemoral amputations using a commercial activity monitor: A feasibility study	Albert, M.V. and Deeny, S. and McCarthy, C. and Valentin, J. and Jayaraman, A.	10.1016/j.pmrj.2014.06.006	2014	USA	[85]
Community activity and participation are reduced in transtibial amputee fallers: A wearable technology study	Hordacre, B. and Barr, C. and Crotty, M.	10.1136/bmjinnov-2014-000014	2015	Australia	[86]
Free-Living Motor Activity Monitoring in Ataxia-Telangiectasia.	Khan NC and Pandey V and Gajos KZ and Gupta AS	10.1007/s12311-021-01306-y	2022	USA	[95]
Real-life ankle submovements and computer mouse use reflect patient-reported function in adult ataxias.	Eklund NM and Ouillon J and Pandey V and Stephen CD and Schmahmann JD and Edgerton J and Gajos KZ and Gupta AS	10.1093/braincomms/fcad064	2023	USA	[96]
Digital endpoints for self-administered home-based functional assessment in pediatric Friedreich's ataxia	Mueller, A and Paterson, E and McIntosh, A and Praestgaard, J and Bylo, M and Hoefling, H and Wells, M and Lynch, DR and Rummey, C and Krishnan, ML and Schultz, M and Malanga, CJ	10.1002/acn3.51438	2021	USA	[100]
Accelerometric Gait Analysis Devices in Children-Will They Accept Them? Results From the AVAPed Study	Wiedmann, I and Grassi, M and Duran, I and Lavrador, R and Alberg, E and Daumer, M and Schoenau, E and Rittweger, J	10.3389/fped.2020.574443	2021	Germany	[93]
Disease-specific wearable sensor algorithms for profiling activity, gait, and balance in individuals with Charcot-Marie-Tooth disease type 1A	Dinesh, K and White, N and Baker, L and Sowden, JE and Behrens-Spraggins, S and Wood, E and Charles, J and Herrmann, DN and Sharma, G and Eichinger, K	10.1111/jns.12562	2023	USA	[91]
The Effect of Pain Relief on Daily Physical Activity: In-Home Objective Physical Activity Assessment in Chronic Low Back	Chuan Yen T and Mohler J and Dohm M and Laksari K and Najafi B and Toosizadeh N	10.3390/s18093048	2018	USA	[97]

Pain Patients after Paravertebral Spinal Block.					
Sensor-Derived Physical Activity Parameters Can Predict Future Falls in People with Dementia	Schwenk, M and Hauer, K and Zieschang, T and Englert, S and Mohler, J and Najafi, B	10.1159/000363136	2014	USA	[99]
Quantification of Motor Function in Huntington Disease Patients Using Wearable Sensor Devices.	Gordon MF and Grachev ID and Mazeh I and Dolan Y and Reilmann R and Loupe PS and Fine S and Navon-Perry L and Gross N and Papapetropoulos S and Savola JM and Hayden MR	10.1159/000502136	2019	Switzerland	[80]
Wearable Sensors in Huntington Disease: A Pilot Study.	Andrzejewski KL and Dowling AV and Stamler D and Felong TJ and Harris DA and Wong C and Cai H and Reilmann R and Little MA and Gwin JT and Biglan KM and Dorsey ER	10.3233/JHD-160197	2016	Netherlands	[82]
A Longitudinal Wearable Sensor Study in Huntington's Disease	Dinesh, K and Snyder, CW and Xiong, ML and Tarolli, CG and Sharma, S and Dorsey, ER and Sharma, G and Adams, JL	10.3233/JHD-190375	2020	USA	[81]
A Remote Digital Monitoring Platform to Assess Cognitive and Motor Symptoms in Huntington Disease: Cross-sectional Validation Study.	Lipsmeier F and Simillion C and Bamdadian A and Tortelli R and Byrne LM and Zhang YP and Wolf D and Smith AV and Czech C and Gossens C et al.	10.2196/32997	2022	Canada	[42]
Detection of Real-World Trips in At-Fall Risk Community Dwelling Older Adults Using Wearable Sensors.	Handelzalts S and Alexander NB and Mastruserio N and Nyquist LV and Strasburg DM and Ojeda LV	10.3389/fmed.2020.00514	2020	Switzerland	[83]
Continuous daily assessment of multiple sclerosis disability using remote step count monitoring	Block, VJ and Lizee, A and Crabtree-Hartman, E and Bevan, CJ and Graves, JS and Bove, R and Green, AJ and Nourbakhsh, B and Tremblay, M and Gourraud, PA et al.	10.1007/s00415-016-8334-6	2017	USA	[43]

Remote Monitoring in the Home Validates Clinical Gait Measures for Multiple Sclerosis.	Supratak A and Datta G and Gafson AR and Nicholas R and Guo Y and Matthews PM	10.3389/fneur.2018.00561	2018	United kingdom	[78]
Open-source dataset reveals relationship between walking bout duration and fall risk classification performance in persons with multiple sclerosis.	Meyer BM and Tulipani LJ and Gurchiek RD and Allen DA and Solomon AJ and Cheney N and McGinnis RS	10.1371/journal.pdig.0000120	2022	USA	[77]
Fall Prediction Based on Instrumented Measures of Gait and Turning in Daily Life in People with Multiple Sclerosis	Arpan, I and Shah, VV and McNames, J and Harker, G and Carlson-Kuhta, P and Spain, R and El-Gohary, M and Mancini, M and Horak, FB	10.3390/s22165940	2022	USA	[79]
Home-based gait analysis as an exploratory endpoint during a multicenter phase 1 trial in limb girdle muscular dystrophy type R2 and facioscapulohumeral muscular dystrophy.	Gidaro T and Gasnier E and Annoussamy M and Vissing J and Attarian S and Mozaffar T and Iyadurai S and Wagner KR and Vissière D and Walker G and Shukla SS and Servais L	10.1002/mus.27446	2022	USA	[104]
Quantifying real-world upper limb activity via patient-initiated spontaneous movement in neonatal brachial plexus palsy	Gatward, ME and Logue, RN and Yang, LJS and Brown, SH	10.1002/pmrj.12780	2023	USA	[92]
Remotely delivered, individualized, and self-directed gait modification for knee osteoarthritis: A pilot trial	Charlton, JM and Krowchuk, NM and Eng, JJ and Li, LC and Hunt, MA	10.1016/j.clinbiomech.2023.105981	2023	Canada	[89]
Observational Study of a Wearable Sensor and Smartphone Application Supporting Unsupervised Exercises to Assess Pain and Stiffness.	Perraudin CGM and Illiano VP and Calvo F and O'Hare E and Donnelly SC and Mullan RH and Sander O and Caulfield B and Dorn JF	10.1159/000493277	2018	Switzerland	[90]
Fall Risk Prediction in Parkinson's Disease Using Real-World Inertial Sensor Gait Data	Ullrich, M and Roth, N and Kuederle, A and Richer, R and Gladow, T and Gassner, H and Marxreiter, F and Klucken, J and Eskofier, BM and Kluge, F	10.1109/JBHI.2022.3215921	2023	Germany	[52]

iTex Gloves: Design and In-Home Evaluation of an E-Textile Glove System for Tele-Assessment of Parkinson's Disease	Ravichandran, V and Sadhu, S and Convey, D and Guerrier, S and Chomal, S and Dupre, AM and Akbar, U and Solanki, D and Mankodiya, K	10.3390/s23062877	2023	USA	[34]
Automated detection of missteps during community ambulation in patients with Parkinson's disease: a new approach for quantifying fall risk in the community setting	Iluz, T and Gazit, E and Herman, T and Sprecher, E and Brozgol, M and Giladi, N and Mirelman, A and Hausdorff, JM	10.1186/1743-0003-11-48	2014	Israel	[46]
Accelerometer data collected with a minimum set of wearable sensors from subjects with Parkinson's disease	Daneault, JF and Vergara-Diaz, G and Parisi, F and Admati, C and Alfonso, C and Bertoli, M and Bonizzoni, E and Carvalho, GF and Costante, G and Fabara, et al.	10.1038/s41597-021-00830-0	2021	USA	[45]
Detection of Unsupervised Standardized Gait Tests From Real-World Inertial Sensor Data in Parkinson's Disease	Ullrich, M and Mucke, A and Kuderle, A and Roth, N and Gladow, T and Gassner, H and Marxreiter, F and Klucken, J and Eskofier, BM and Kluge, F	10.1109/TNSRE.2021.3119390	2021	Germany	[47]
Monitoring Parkinson's disease symptoms during daily life: a feasibility study	Heijmans, M and Habets, JGV and Herff, C and Aarts, J and Stevens, A and Kuijf, ML and Kubben, PL	10.1038/s41531-019-0093-5	2019	Netherlands	[49]
Measuring freezing of gait during daily-life: an open-source, wearable sensors approach	Mancini, M. and Shah, V.V. and Stuart, S. and Curtze, C. and Horak, F.B. and Safarpour, D. and Nutt, J.G.	10.1186/s12984-020-00774-3	2021	USA	[51]
Limb and trunk accelerometer data collected with wearable sensors from subjects with Parkinson's disease.	Vergara-Diaz G and Daneault JF and Parisi F and Admati C and Alfonso C and Bertoli M and Bonizzoni E and Carvalho GF and Costante G and Fabara EE et al	10.1038/s41597-021-00831-z	2021	USA	[48]
Feasibility of a wearable inertial sensor to assess motor complications and treatment in Parkinson's disease	Caballol, N and Bayes, A and Prats, A and Martin-Baranera, M and Quispe, P	10.1371/journal.pone.0279910	2023	Spain	[50]

Virtual exam for Parkinson's disease enables frequent and reliable remote measurements of motor function	Burq, M and Rainaldi, E and Ho, KC and Chen, C and Bloem, BR and Evers, LJW and Helmich, RC and Myers, L and Marks, W and Kapur, R	10.1038/s41746-022-00607-8	2022	USA	[37]
A fuzzy logic system for the home assessment of freezing of gait in subjects with Parkinsons disease	Pepa, L and Capecci, M and Andrenelli, E and Ciabattoni, L and Spalazzi, L and Ceravolo, MG	10.1016/j.eswa.2020.113197	2020	Italy	[44]
A "HOLTER" for Parkinson's disease: Validation of the ability to detect on-off states using the REMPARK system	Bayes, A and Samba, A and Prats, A and Perez-Lopez, C and Crespo-Maraver, M and Moreno, JM and Alcaine, S and Rodriguez-Molinero, A and Mestre, B and Quispe, P and de Barroset al	10.1016/j.gaitpost.2017.09.031	2018	Spain/Ireland	[53]
Multiple wearable sensors in parkinson and huntington disease individuals: A pilot study in clinic and at home	Adams, J.L. and Dinesh, K. and Xiong, M. and Tarolli, C.G. and Sharma, S. and Sheth, N. and Aranyosi, A.J. and Zhu, W. and Goldenthal, S. and Biglan, K.M. and Dorsey, E.R. and Sharma, G.	10.1159/000479018	2017	USA	[70]
A Wearable Assistant for Gait Training for Parkinson's Disease with Freezing of Gait in Out-of-the-Lab Environments	Mazilu, S and Blanke, U and Dorfman, M and Gazit, E and Mirelman, A and Hausdorff, JM and Troster, G	10.1145/2701431	2015	Belgium	[54]
Acceptability of an In-home Multimodal Sensor Platform for Parkinson Disease: Nonrandomized Qualitative Study	Morgan, C and Tonkin, EL and Craddock, I and Whone, AL	10.2196/36370	2022	United Kingdom	[61]
Could In-Home Sensors Surpass Human Observation of People with Parkinson's at High Risk of Falling? An Ethnographic Study	Stack, E and King, R and Janko, B and Burnett, M and Hammersley, N and Agarwal, V and Hannuna, S and Burrows, A and Ashburn, A	10.1155/2016/3703745	2016	United Kingdom	[55]

Using a smartwatch and smartphone to assess early Parkinson's disease in the WATCH-PD study.	Adams JL and Kangarloo T and Tracey B and O'Donnell P and Volfson D and Latzman RD and Zach N and Alexander R and Bergethon P and Cosman J et al	10.1038/s41531-023-00497-x	2023	USA	[41]
Parkinson's Disease Tremor Detection in the Wild Using Wearable Accelerometers	San-Segundo, R and Zhang, A and Cebulla, A and Panev, S and Tabor, G and Stebbins, K and Massa, RE and Whitford, A and de la Torre, F and Hodgins, J et al	10.3390/s20205817	2020	USA	[57]
Turn Around Freezing: Community-Living Turning Behavior in People with Parkinson's Disease.	Mancini M and Weiss A and Herman T and Hausdorff JM	10.3389/fneur.2018.00018	2018	USA	[40]
Home-Based Physical Behavior in Late Stage Parkinson Disease Dementia: Differences between Cognitive Subtypes	Cerff, B and Maetzler, W and Sulzer, P and Kampmeyer, M and Prinzen, J and Hobert, MA and Blum, D and van Lummel, R and Del Din, S and Graber, S et al	10.1159/000460251	2017	Germany	[67]
Ambulatory surface electromyography with accelerometry for evaluating daily motor fluctuations in Parkinson's disease	Rissanen, SM and Koivu, M and Hartikainen, P and Pekkonen, E	10.1016/j.clinph.2020.11.039	2021	Finnland	[62]
Daily-Living Freezing of Gait as Quantified Using Wearables in People With Parkinson Disease: Comparison With Self-Report and Provocation Tests	Denk, D and Herman, T and Zoetewei, D and Ginis, P and Brozgol, M and Thumm, PC and Decaluwe, E and Ganz, N and Palmerini, L and Giladi, N and Nieuwboer, A and Hausdorff, JM	10.1093/ptj/pzac129	2022	Israel	[56]
Telehealth Management of Parkinson's Disease Using Wearable Sensors: An Exploratory Study.	Heldman DA and Harris DA and Felong T and Andrzejewski KL and Dorsey ER and Giuffrida JP and Goldberg B and Burack MA	10.1159/000475801	2017	USA	[60]

Surrogates for rigidity and PIGD MDS-UPDRS subscores using wearable sensors	Safarpour, D and Dale, ML and Shah, VV and Talman, L and Carlson-Kuhta, P and Horak, FB and Mancini, M	10.1016/j.gaitpost.2021.10.029	2022	USA	[58]
Continuous monitoring of turning in Parkinson's disease: Rehabilitation potential	Mancini, M and El-Gohary, M and Pearson, S and McNames, J and Schlueter, H and Nutt, JG and King, LA and Horak, FB	10.3233/NRE-151236	2015	USA	[63]
Effect of Levodopa and Environmental Setting on Gait and Turning Digital Markers Related to Falls in People with Parkinson's Disease	Shah, VV and McNames, J and Carlson-Kuhta, P and Nutt, JG and El-Gohary, M and Sowalsky, K and Mancini, M and Horak, FB	10.1002/mdc3.13601	2023	USA	[66]
How Time Rules: Diurnal Motor Patterns in de novo Parkinson's Disease.	van Wamelen DJ and Urso D and Ray Chaudhuri K	10.3233/JPD-202352	2021	United Kingdom	[68]
Feasibility of a Multimodal Telemedical Intervention for Patients with Parkinson's Disease-A Pilot Study	Bendig, J and Wolf, AS and Mark, T and Frank, A and Mathiebe, J and Scheibe, M and Mueller, G and Stahr, M and Schmitt, J and Reichmann, H and Loewenbrueck, KF and Falkenburger, BH	10.3390/jcm11041074	2022	Germany	[105]
Home-based monitoring of falls using wearable sensors in Parkinson's disease	de Lima, ALS and Smits, T and Darweesh, SKL and Valenti, G and Milosevic, M and Pijl, M and Baldus, H and de Vries, NM and Meinders, MJ and Bloem, BR	10.1002/mds.27830	2020	Netherlands	[28]
Effect of Fear of Falling on Mobility Measured During Lab and Daily Activity Assessments in Parkinson's Disease.	Atrsaei A and Hansen C and Elshehabi M and Solbrig S and Berg D and Liepelt-Scarfone I and Maetzler W and Aminian K	10.3389/fnagi.2021.722830	2021	Switzerland	[64]
Effect of Fear of Falling on Turning Performance in Parkinson's Disease in the Lab and at Home	Haertner, L and Elshehabi, M and Zaunbrecher, L and Pham, MH and Maetzler, C and van Uem, JMT and Hobert, MA and Hucker, S and Nussbaum, S and	10.3389/fnagi.2018.00078	2018	Germany	[65]

	Berg, D and Liepelt-Scarfone, I and Maetzler, W				
Remote smartphone gait monitoring and fall prediction in Parkinson's disease during the COVID-19 lockdown.	Marano M and Motolese F and Rossi M and Magliozzi A and Yekutieli Z and Di Lazzaro V	10.1007/s10072-021-05351-7	2021	Italy	[69]
A training approach to improve stepping automaticity while dual-tasking in Parkinson's disease: A prospective pilot study.	Chomiak T and Watts A and Meyer N and Pereira FV and Hu B	10.1097/MD.0000000000005934	2017	USA	[59]
Feasibility of large-scale deployment of multiple wearable sensors in Parkinson's disease	de Lima, ALS and Hahn, T and Evers, LJW and de Vries, NM and Cohen, E and Afek, M and Bataille, L and Daeschler, M and Claes, K and Boroojerdi, B and Terricabras, D and Little, MA and Baldus, H and Bloem, BR and Faber, MJ	10.1371/journal.pone.0189161	2017	USA	[38]
Impact of motor fluctuations on real-life gait in Parkinson's patients.	Silva de Lima AL and Evers LJW and Hahn T and de Vries NM and Daeschler M and Boroojerdi B and Terricabras D and Little MA and Bloem BR and Faber MJ	10.1016/j.gaitpost.2018.03.045	2018	Netherlands	[39]
TELEREHABILITATION OF THE KNEE JOINTS OF PATIENTS WITH POLYTRAUMA	Tsvyakh, A.I. and Hospodarskyy, A.Y. and Marchenkova, N.O. and Kopytchak, I.R. and Kostjuk, V.P. and Lymar, Y.A. and Gdanskyi, S.M.	10.36740/wlek202101109	2021	Ukraine	[98]
Wearable monitoring of positive and negative myoclonus in progressive myoclonic epilepsy type 1	Rissanen, SM and Hypponen, J and Silvennoinen, K and Saisanen, L and Karjalainen, PA and Mervaala, E and Kalviainen, R	10.1016/j.clinph.2021.06.026	2021	Finnland	[62]

Reliability of Wearable-Sensor-Derived Measures of Physical Activity in Wheelchair-Dependent Spinal Cord Injured Patients.	Schneider S and Popp WL and Brogioli M and Albisser U and Demkó L and Debecker I and Velstra IM and Gassert R and Curt A	10.3389/fneur.2018.01039	2018	Switzerland	[103]
Physical Activity Monitoring Using a Fitbit Device in Ischemic Stroke Patients: Prospective Cohort Feasibility Study.	Katzan I and Schuster A and Kinzy T	10.2196/14494	2021	USA	[71]
Quantification of the relative arm use in patients with hemiparesis using inertial measurement units.	David A and ReethaJanetSureka S and Gayathri S and Annamalai SJ and Samuelkamleshkumar S and Kuruvilla A and Magimairaj HP and Varadhan S and Balasubramanian S	10.1177/20556683211019694	2021	United Kingdom	[72]
The Feasibility and Longitudinal Effects of a Home-Based Sedentary Behavior Change Intervention After Stroke	Ezeugwu, V.E. and Manns, P.J.	10.1016/j.apmr.2018.06.014	2018	Canada	[75]
Predicting daily use of the affected upper extremity 1 year after stroke	Rand, D. and Eng, J.J.	10.1016/j.jstrokecerebrovasdis.2014.07.039	2015	Canada	[74]
Usability of a wearable device for home-based upper limb telerehabilitation in persons with stroke: A mixed-methods study	Toh, SFM and Gonzalez, PC and Fong, KNK	10.1177/20552076231153737	2023	Hong Kong	[76]
A Longitudinal Investigation of the Efficacy of Supported In-Home Post-Stroke Rehabilitation	Fang, Q and Mahmoud, SS and Kumar, A and Gu, XD and Fu, JM	10.1109/ACCESS.2020.3010674	2020	China	[73]

Part 2 of 3: Data extraction on on studies' objectives, methods and main results

Reference number	Aims	Study Design	Population	Intervention group	Sample size	Dropout or not analysed	Control group (n.)	Control group (feature)	Name of the device	Sensor type	Number of sensors worn	Location	Endpoints	System's validation	Target	Outcome measure	Monitoring (days)	Feasability assessment [Compliance AND /OR Acceptability	Main Results	Accuracy/Reliability/ Acceptability/Compliance	Clinical outcome results
**[102]**	"to examine mobility characteristics of a small group of 12 older adults with ADRD and mild cognitive impairment in their homes"	Case series	Alzheimer's disease	12	12				Opal	tri-axial accelerometer, tri-axial gyroscope, tri-axial magnetometer	1	lower back	"to assess the feasibility of using objective characteristics of turning quality in real-life conditions as a measure of wandering behavior."	against in-clinic assessment	Turning events	Turning events (lasting between 0.5 and 10 s with turn angles of at least 45 degrees)	7	Accuracy/ Reliability	"Comparing our data to Mancini et al.'s study of older adults with and without cognitive impairment, our cohort of participants with cognitive impairment trended toward having a greater number of turns in 30 min, a shorter mean turn duration, a faster mean peak turning speed, and a smaller mean turn angle".	Accuracy: yes	greater number of turns in 30 min, a shorter mean turn duration, a faster mean peak turning speed, and a smaller mean turn angle
**[94]**	"to determine the feasibility of conducting clinical and free-living gait assessments in a dementia population"	cohort study	Alzheimer's disease	16	66		50	Healthy	Axivity AX3	tri-axial accelerometer	1	lower back	"correlation between global cognition and step velocity, step lenght, step velocity		Gait parameters		4	n.a.	"strong negative correlations between global cognition and step lenght,step velocity and number of bouts. Strong		Correlations between global cognition and step lenght,step velocity and number of bouts:

												variability, number daily bouts."						positive correlations between global cognition and step velocity variability."		strong negative. Correlations between global cognition and step velocity variability: Strong positive
**[84]**	"Describe a novel method for activity monitoring and use it to count steps taken while walking straight ahead on level ground, turning right and left, up and down stairs, and up and down ramps."	Case series	Amputee	10	10			not specified	tri-axial accelerometer, tri-axial gyroscope	1	prosthetic pylon	"steps taken while walking straight, turning right, turning left, ramp down, ramp up, stair down, and stair up, turning with the prosthetic leg on the inside and the turning with the non-amputated leg on inside."	against gold standars	Turning events, gait parameters	Turn (angular displacement in the transverse plane, angular displacement of the pylon in the sagittal plane), gait cycle	7	Accuracy/ Reliability	"Overall classification accuracy was 97.5±1.5%. When participants were free to walk about their home, work, and community environments, 82.8% of all steps were in a straight line, 9.0% were turning steps, 4.8% were steps on stairs, and 3.6% were steps on ramps."	Accuracy: 97.5±1.5%	82.8% of all steps were in a straight line, 9.0% were turning steps, 4.8% were steps on stairs, and 3.6% were steps on ramps

**[33]**	"Developing and testing a technology to monitor bodily position and type of activity in a group of transtibial prosthesis users"	cohort study	Amputee	4	4				WAFER	coil antenna	4	prosthetic pylon	Sits, seated shifts, stands, standing weight-shifts, walks, partial doffs, and non-use (full doffs) detections accuracies.	against in-clinic assessment	Quantity of movement	algorithm to detect walking bouts, sits, stands	14	Accuracy/ Reliability	"Sit and walk detection accuracies were above 95% for all four participants tested. Stand detection accuracy was above 90% for three participants and 62.5% for one participant. Step count was not proportional to active use time (sum of stand, walk, and standing weight-shift times)."	Accuracy: Sit and walk 95%, stand 90% for three participanti, 62,5% for one.	Step count not proportional to active use time
**[88]**	The purpose of this study was to explore the clinical viability of using a system of IMU and GPS sensors to characterize the functional mobility of people with LLA. We measured cadence, walking speed, characteristics of their distributions, and how they change with location. We	cohort study	Amputee	16	31	1	14	Healthy	ActiGraph GT9X	tri-axial accelerometer	2	prosthetic pylon	changes in cadence, walking speed and group differences in walkiing sped and stride lenght - not specified primary or secondary outcome		Gait parameters	cadence, walking speed, stride lenght	2	n.a.	The findings can be summarized as follows: (1) both healthy controls and individuals with LLA walked slower during their daily lives compared to their inlab measures (2) cadence variance during daily life was not different between the control and LLA groups, and (3) both groups		Both healthy controls and individuals with LLA walked slower during their daily lives compared to their inlab measures (2) cadence variance during daily life was not different between

used wearable sensors to measure patient walking during their daily lives to provide a better understanding of everyday walking performance, in addition to in-lab capacity measures. Our secondary goal was to examine the potential added value of "everyday performance measures" as compared with in-lab measures of capacity. Because everyday cadence variability and walking speed have yet to be quantified using this method, we also recruited healthy, non-amputee adults to provide a basis for comparison	walked significantly faster outside of the home	the control and LLA groups, and (3) both groups walked significantly faster outside of the home

**[87]**	"To determine whether mean daily step count changed between in-patient rehabilitation and consecutive leave periods."	Case series	Amputee	9	9	ActivPal	tri-axial accelerometer	1	prosthetic pylon	"1) to characterise the daily step count levels of military personnel with bilateral lower limb amputations, 2) to compare the step count levels during and between in-patient rehabilitation intervals."	Quantity of movement	unilateral monitor-side steps per day'	7	n.a.	"Mean daily step count significantly decreased from 2258±192 during in-patient rehabilitation to 1387±363 at home"	Mean daily step count significantly decreased from 2258±192 during in-patient rehabilitation to 1387±363 at home
**[85]**	"To assess in a feasibility study the mobility of persons with transfemoralamputations using data collected from a popular, consumer-oriented activity monitor"	Case series	Amputee	9	9	Fitbit One	tri-axial accelerometer	1	wrist	"Daily estimates of step counts, distance walked,floors/stairs climbed, calories burned, and proprietary Fitbit activity scores. For each day, theamount of time in each of the following levels of activity was also	Quantity of movement	Gait (step counts, distance walked, floors climbed, calories burned, and Fitbitactivity scores), Fitbit Activity Level (amount of time in each kind of daily	7	n.a.	"Estimated step counts were highlypredictive/redundant with estimated miles walked without setting individual stride lengths"	Estimated step counts: highly predictive/redundant with estimated miles walked without setting individual stride lengths

											reported: sedentary ,lightly active, fairly active, and highly active."		activity)				
**[86]**	"to assess activity and participation characteristics in the home and various community settings for transtibial amputee fallers and non-fallers"	Case series	Amputee	46	47	1	StepWatch activity monitor (SAM)	tri-axial accelerometer, GPS travel recorder	1	wrist	"Community activity and home activity"	Quantity of movement	latitude and longitude data (GPS)	14	n.a.	"Participants with a history of falls demonstrated significantly lower levels of community activity (p=0.01) and participation (p=0.02). Specifically, activity levels were reduced for recreational (p=0.01) and commercial roles (p=0.02), while participation was lower for recreational roles (p=0.04)."	Fallers: activity levels reduced for recreational (p=0.01) and commercial roles (p=0.02), while participation was lower for recreational roles (p=0.04)

**[95]**	"To test the hypothesis that metrics derived from a single wrist sensor worn at home provide accurate, reliable, and interpretable information about neurological disease severity in children with Ataxia-telangiectasia (A-T.)"	cohort study	Ataxia	15	30	15	Healthy	GeneActiv	tri-axial accelerometer	1	wrist	"Activity Index (AI) and his Correlation with Neurological Severity using the Brief Ataxia Rating Scale (BARS), Reliability of Wearable Sensor Measures, reliability and influence of age on wearable sensors data, Relationship with Task-Based Digital Measures "	Scale/index/test correlation, symptom		7	Accuracy/ Reliability	" Children with A-T were inactive the same proportion of each day as controls but produced more low intensity movements (p < 0.01; Cohen's d = 1.48) and fewer high intensity movements (p < 0.001; Cohen's d = 1.71). The range of activity intensities was markedly reduced in A-T compared to controls (p < 0.0001; Cohen's d = 2.72)"	Reliability: The activity metrics correlated strongly with arm, gait, and total clinical severity (r: 0.71-0.87; p < 0.0001),	Children with A-T: low intensity movements (p < 0.01; Cohen's d = 1.48), fewer high intensity movements (p < 0.001; Cohen's d = 1.71). The range of activity intensities was markedly reduced in A-T compared to controls (p < 0.0001; Cohen's d = 2.72
**[96]**	"to test if sensors worn continuously at home during natural behaviour and a web-based computer mouse task performed at home could produce interpretable, meaningful	cohort study	Ataxia	34	42	8	Healthy	GeneActiv	tri-axial accelerometer	2	ankle, wrist	"ankle submovements, ankle submovements correlation with ataxia rating scale scores and patient's self-	Scale/index/test correlation, quantity of move	Ankle and wrist sensor data (These included Activity intensity (AI) mean (one feature), AI	7	n.a.	"Individuals with ataxia had smaller, slower and less powerful ankle submovements during natural behaviour at home. A composite measure based on ankle submovemen		Individuals with ataxia: smaller, slower and less powerful ankle submovements during natural behaviour at home. A composit

	and reliable motor measures for potential use in clinical trials"											reported function"	ment	entropy (one feature), Submovement (SM) distance (eight features), SM velocity (eight features) and SM acceleration (eight features)			ts strongly correlated with ataxia rating scale scores, strongly correlated with self-reported function."	e measure based on ankle submovements strongly correlated with ataxia rating scale scores, strongly correlated with self-reported function
**[100]**	"to valuate the utility of home-based, self-administered digital endpoints in children with Friedreich's ataxia"	cohort study	Ataxia	13	25	12	Healthy	PhysiLog 5	tri-axial accelerometer, tri-axial gyroscope	5	feet, wrists, trunk	"Gait and balance assessment"	Symptom, gait parameters	Gait (swing and stance periods)	6	n.a.	"The control group was significantly more active than the FA group with foot and wrist movements (both P < 0.01) but not with trunk movements. The FA group spent more time in stance phase and had high peak acceleration during swing time."	Control group: significantly more active than the FA group with foot and wrist movements (both P < 0.01) but not with trunk movements. FA group: more time spent in stance phase, high peak acceleration during swing time

**[93]**	"To assess children's acceptance to wear a 3D-accelerometer, to compare gait speed during supervised testing with the non-supervised gait speed in every-day life." "to validate 3D-accelerometric assessment against a gold standard method"	cohort study	Cerebral Palsy	30	60	30	Healthy	Actibelt® RCT 2	tri-axial accelerometer	1	waist	"children's acceptance to wear a 3D-accelerometer and compare gait speed during supervised testing with the non-supervised gait speed in every-day life" "to validate 3D-accelerometric assessment against a gold standard method"	against gold standars	Gait parameters	wearing time, questionnaire wearing comfort, gait speed	7	Compliance	"Wearing time amounted to 10.3 hours per day, 3D-accelerometry is well-enough accepted in a pediatric population of patients with Celebral palsy." "The assessment of habitual activities by wearable devices reflects the functioning of children in their home environment."	Compliance: 10,3 hours (well-enough accepted) of wearing time	wearable devices reflects the functioning of children in their home environmen
**[91]**	"to provide useful outcome measures for future trials using wearable sensors"	cohort study	Charcot-Marie-Tooth Disease type 1A (CMT1A) patients	15	30	15	Healthy	BioStamp RC	tri-axial accelerometer	3	chest, thigh, tibia	" activity, gait, and balance parameters"	against in-clinic assessment	Symptom, gait parameters, quantity of movement	Trunk data (from the trunk and thigh sensors). Gait: (step count, step duration, step length, and gait speed). Balance: (Sway jerk and	1	Accuracy/ Reliability	"CMT1A participants had lon ger step durations (p < .001), shorter step lengths (p = .03), slower gait speeds (p < .001), and greater postural sway (p < .001) than healthy controls"	Reliability: Gait and balance metrics demonstrated moderate to excellent reliability	CMT1A: longer step durations (p < .001), shorter step lengths (p = .03), slower gait speeds (p < .001), and greater postural sway (p < .001) than healthy controls

															sway area.)					
**[97]**	"to investigate the efficacy of paravertebral facet injection for degenerative facet osteoarthropathy,"	Case series	Degenerative facet osteoartropathy	8	8			PAM Sys	tri-axial accelerometer	1	mid-sternal	"gait speed, gait cycle time, and total Timed Up and Go (TUG) duration comparison between pre-injection period, post-injection and a month post-injection."		Gait parameters	Gait speed, gait cycle, stride lenght, speed variability, time spent for a TUG test	6	n.a.	"gait and TUG were significantly improved (~16% and ~18% faster walking and shorter TUG, $p < 0.05$); however, DPA, including the percentage of physical activities (walking and standing) and the number of steps, showed no significant change after PSI ($p > 0.25$; effect size ≤ 0.44). "		Gait and TUG: significantly improved (~16% and ~18% faster walking and shorter TUG, $p < 0.05$); percentage of physical activities (walking and standing) and the number of steps: no significant change after PSI ($p > 0.25$; effect size ≤ 0.44).
**[99]**	"to explore the validity of sensor-derived physical activity (PA) in people with dementia"	cohort study	Dementia	28	77	49	Dementia	Physilog	tri-axial accelerometer, tri-axial gyroscope	1	chest	"walking, standing, sitting and lying."	against in-clinic assessment	Quantyt of movement, gait param	walking, standing, sitting, lying during 24h, walking bouts	1	Accuracy/ Reliability	" The 'walking bout average duration', 'longest walking bout duration' and 'walking bout duration variability' were lower in	Sensitivity: 80% Specificity: 55%	Fallers: lower walking bout average duration and walking bout duration variability (p =

														eters				fallers, compared to non-fallers (p = 0.008-0.027). The ‘standing bout average duration' was higher in fallers (p = 0.050). "		0.008-0.027). Higher standing bout average duration (p = 0.050).
**[80]**	"to develop algorithms that could objectively quantify chorea by analyzing the data from the wearable sensors, to ultimately increase the clinical understanding of Huntingtond disease (HD), and to provide insight into the daily variability of HD motor symptoms outside the clinical setting."	Case series	Huntington's chorea	10	15	5		Smartphone , smartwatch (Customized app)	tri-axial accelerometer, tri-axial gyroscope	2	arm, wrist	"Chorea severity score, model performance"	against in-clinic assessment, patient-reported	Score/Index/test correlation, symptom	algorithm on whether or not chorea exists (based on the clinician ranking of chorea severity)	6	Accuracy/ Reliability	"the sensor-based model can quantify the chorea level with high correlation to the chorea severity reported by both clinicians and patients. In addition, our analysis shows that the chorea digital signature varies between patients."	Reliability: high correlation between algorithm and chorea severity score reported by both clinicians and patients. sensitivity = 85%, specificity = 72%, positive predictive value (precision) = 90%, accuracy = 81%, and negative predictive value = 62%.	
**[82]**	To evaluate the feasibility and ability of wearable sensors to measure motor impairment in individuals with Huntington	cohort study	Huntington's chorea	15	20	5	Healthy	PAM Sys X	tri-axial accelerometer	5	chest, ankles, wrists	Feasibility, changes of physical activities, gait		Quantity of movement, Gait Par	feasibility, physical activity at home, gait analysis	180	Accuracy/ Reliability		Accuracy: yes	At home with additional observations, significant differences were observed

	disease in the clinic and at home.	ameters	in seven additional gait measures. The gait of individuals with higher total motor scores (50 or more) differed significantly from those with lower total motor scores (below 50) on multiple measures at home.

**[81]**	"To measure motor symptoms of Huntington's Disease (HD) using wearable sensors"	randomized controlled trial	Huntington's chorea	20	39	19	Healthy	BioStamp RC	tri-axial accelerometer	5	chest, thighs, arms	"To evaluate the mean differences in the truncal Chorea Index, gait, and activity parameters between groups and assess the longitudinal progression within groups"	against in-clinic assessment	Scale/index/test correlation, Gait parameters	Chorea: raw sensors. Gait: step count, step duration, step length, walk speed, and coordination between the legs. Daily activities: based on the combination of dominant acceleration axes of the trunk and thigh sensors.	28	Accuracy/ Reliability	The average truncal Chorea Index was higher in individuals with HD (26.6, p < 0.001) than in controls (15.6). For participants with HD, the truncal Chorea Index showed a high intra-day variability but minimal change over 12 months. Individuals with HD walked less (HD = 3818, prodromal HD = 6957, controls = 5514 steps/day) and took longer duration steps (HD = 0.97, prodromal HD = 0.78, controls = 0.85 seconds/step) than the other groups. Individuals with HD spent over half their day lying down (HD = 51.1%,	Reliability: the p-value for the in-clinic duration falls just above the traditional 5% significance level	HD patients: higher average truncal chorea index, higher intra-day variability but minimal 12 months variation, walked less, longer step duration.

																	prodromal HD = 38.0%, controls = 37.1%).	
**[42]**	"to assess motor, cognitive, behavioral, and functional domains in HD using frequent active and continuous passive monitoring"	Case series	Huntington's chorea	218	219	1	Smartphone (Galaxy J7; Samsung), smartwatch (Moto G 360 2nd Gen Sport; Motorola), App name Roche HD	tri-axial accelerometer, tri-axial gyroscope	2	wrist, arm	feasibility, accuracy."	against in-clinic assessment	Score/index/test	wearing time, comparison between system and clinical measures	2	Accuracy/ Reliability	"All sensor-based features showed good to excellent test-retest reliability (intraclass correlation coefficient 0.89-0.98)"	test-retest reliability: good to excellent (intraclass correlation coefficient 0.89-0.98)

**[83]**	"to explore key body kinematics underlying real-world trips in at-fall risk community dwelling older adults wearing inertial measurement units (IMU)"	Case series	Loss of balance	5	5		Opal	tri-axial accelerometer, tri-axial gyroscope, tri-axial magnetometer	4	feet, lower back, wrist	"feasibility on detecting foot velocity signal during swing phase; velocity of contralateral foot and sharp changes in lower back pitch angles" (all of them associated to "trip" events like "stubbled foot")	against patient-reported	Gait parameters	foot velocity, lower back pith angles	14	Accuracy/ Reliability	"Our approach demonstrates the feasibility of identifying and studying the mechanisms and context underlying trip-related LOBs in at-fall risk older adults during real world activities."	Accuracy on identifying "trip" events related to less of balance: yes	
**[43]**	"to determine whether remote step count monitoring using a consumer-friendly accelerometer (Fitbit Flex) can enhance MS disability assessment"	Case series	Multiple Sclerosis	93	99	6	Fitbit Flex	tri-axial accelerometer	1	wrist	"1) To determine whether remote step count monitoring can enhance Multiple Sclerosis disability assessment and 2) to determine the validity of the Fitbit Flex as an economical,	against gold standars	Quantity of movement	step count	7	Accuracy/ Reliability	" Substudy validation resulted in high interclass correlations between Fitbit, ActiGraph and manual step count tally and between Fitbit and ActiGraph (ICC = 0.76) during 7-day home monitoring. Over 4 weeks of continuous monitoring, daily steps	high interclass correlations between Fitbit, ActiGraph and manual step count tally and between Fitbit and ActiGraph (ICC = 0.76)	daily steps were lower in progressive versus relapsing Multiple Scleroris

												consumer-friendly alternative to a commonly used research-grade activity monitor (ActiGraph)"						were lower in progressive versus relapsing MS."		
**[78]**	"to measure walking speed remotely in the home environment"	cohort study	Multiple Sclerosis	32	39	7	Healthy	Axivity AX3	tri-axial accelerometer	1	lower back	"prediction of gait speed based on timed 25 foot walk (T25W) clinic testing, accuracy of the model."	against in-clinic assessment	Scale/Index/test correlation, gait parameter	Acceleration data for walking speed estimation	28	Accuracy/ Reliability	"the accuracy of individually personalized models was high regardless of disability. With the latter, we confirmed that the clinic T25FW is strongly predictive of the maximum sustained gait speed in the home environment."	Accuracy: high regardless of disability	
**[77]**	"to perform a remote gait analysis and use it to examine how gait parameters and fall risk classification performance change in relation to walking bout duration in Patients with	Case series	Multiple Sclerosis	38	38			BioStamp RC	tri-axial accelerometer	2	chest, thigh	"Gait bouts, stride detection, Gait parameter data, fall risk classification."	against in-clinic assessment	Fall, Gait parameters	step and stride frequency, duration of the walking bout,	2	Accuracy/ Reliability	"short duration free-living walking bouts were found to be the least similar to laboratory walking, longer duration free-living walking bouts	Fall classification models trained on the gait parameters explored in this study performed best on lab walking bouts and short walking	short duration free-living walking bouts: the least similar to laboratory walking. Longer duration free-living

	Multiple Sclerosis (MS)."																	provided more significant differences between fallers and non-fallers, and an aggregation of all free-living walking bouts yields the best performance in fall risk classification. "	bouts when considering home walking only.	walking bouts: more significant differences between fallers and non-fallers. An aggregation of all free-living walking bouts: yields the best performance in fall risk classification.
**[79]**	" to study the potential of passive monitoring of gait and turning in daily life in people with multiple sclerosis (PwMS) to identify those at future risk of falls."	cohort study	Multiple Sclerosis	13	26		13	Multiple sclerosis	Opal	tri-axial accelerometer, tri-axial gyroscope, tri-axial magnetometer	3	waist, socks	"Gait and turning measures during daily life, falling monitoring"		Turining events, gait parameters		7	n.a.	"Among quantitative monitoring measures of gait and turning, the pitch at toe-off was the best predictor of falls (AUC = 0.86, p < 0.01). Fallers had a smaller pitch of their feet at toe-off, reflecting less plantarflexion during the push-off phase of walking."		The pitch at toe-off was the best predictor of falls (AUC = 0.86, p < 0.01). Fallers: smaller pitch of their feet at toe-off

**[104]**	"to evaluate potential digital outcome recorded by WMIS in ambulant Limb girdle muscular dystrophy type (2BLGMDR2) and acioscapulohumeral muscular dystrophy (FSHD) patients"	Case series	Muscolar Dystrophy	10	18	8			ActiMyo®	tri-axial accelerometer, tri-axial gyroscope, tri-axial magnetometer, barometer	2	wrist, ankle	"cumulative distance walked, the total number of stride, stride length and the stride speed"		Quantity of movement, gait parameters	Gait detection (cumulative distance walked, the total number of stride, stride length and the stride speed)	30	n.a.	" Longitudinal analyses showed a slight but significant decrease in stride speed at month 4 for all subjects. Activity variables such as total number of strides per day were highly variable from month to month in individual patients, and no visit effects were found for thisvariable"		Stride speed at month 4: slight but significant decrease in for all subjects. total number of strides per day:highly variable from month to month in individual patients, no visit effects were found for this variable
**[92]**	"To determine the feasibility of using body-worn accelerometers to remotely assess arm movements in children with Neonatal Brachial plexus palsy (NBPP)."	cohort study	Neonatal brachial plexus palsy (adolescents)	9	18		9	Healthy	ActiGraph GT9X	tri-axial accelerometer	2	wrists	"ratios of affected to unaffected arm motion for duration and magnitude"	against in-clinic assessment	Quantity of movement	magnitude and ratios of affected to unaffected arm motion	7	Accuracy/ Reliability	"duration of arm movement and magnituderatios were reduced in the NBPP group, particularly for arm magnitude due to reduced affected arm movement and an increase in unaffected arm movement."	Reliability: Mallet scores were strongly correlated with both Vector Magnitude and Vector Time indicating that greater shoulder function was predictive of greater self-initiated movement of the arm.	Arm movement duration and magnitude ratios: reduced in the NBPP group (particularly for arm magnitude)

**[89]**	"to assess a remotely delivered and self-directed gait modification for knee osteoarthritis"	randomized controlled trial	osteoarthritis	10	20	10	Osteoarthritis	not specified	tri-axial accelerometer, tri-axial gyroscope, tri-axial magnetometer	1	shoe	"feasibility, adherence, pain, Knee Injury and Osteoarthritis Outcome Scale (KOOS); knee joint moment magnitudes including knee adduction moment (KAM), the KAM magnitudes at the first (KAM1) and second (KAM2) peak of the vertical ground reaction force, and the peak and impulse of the knee flexion moment (KFMp, and KFMi, respectively).	against in-clinic assessment	Quantity of movement	algorithm parameters, pain questionnaire	42	Acceptability	"By follow up, participants reported high confidence (8.6/10), low difficulty (2.0/10), and satisfaction (75%) with the intervention and no significant adverse events. Foot progression angle was modified by 11.4° ± 5.6, which was significantly different ($p < 0.001$, $\eta 2g = 0.8$) when compared between groups. No other between-group differences were significant, while several significant pre-post improvements in pain ($d = 0.6$, $p = 0.006$) and knee moments ($d = 0.6$, $p = 0.01$) were observed."	Acceptancy: high confidence (8.6/10), low difficulty (2.0/10), and satisfaction (75%) with the intervention and no significant adverse events.	Foot progression angle was modified by 11.4° ± 5.6, which was significantly different ($p < 0.001$, $\eta 2g = 0.8$) when compared between groups. Several significant pre-post improvements in pain ($d = 0.6$, $p = 0.006$) and knee moments ($d = 0.6$, $p = 0.01$) were observed

**[90]**	"to explore whether the participants would perform the test 5xSTS (5 times sit to stand) regularly in their home environment, and do so in a correct and consistent manner, to demonstrate that the measurements collected would enable us to derive an objective signal related to morning pain and stiffness."	cohort study	osteoarthritis-rheumatoid arthritis-psoriatic arthritis	30	45	15	Healthy	ActiGraph GT9X	tri-axial accelerometer	2	wrists	"Participants' Adherence, Consistency of 5×STS Execution and his relation with pain and stiffness."	against patient-reported	Score/Index/test correlation, symptom	wearing time, accelerometry traces within and between participants, duration of the 5×Sit To Stand test	28	Compliance	"The participants performed 56% of the prescribed 5×STS tests. We showed that 5×STS test duration (the time taken to complete the 5×STS test) was significantly and robustly associated with the pain and stiffness intensity reported via the PROs"	Compliance: The participants performed 56% of the prescribed 5×STS tests	5×STS test duration: significantly and robustly associated with the pain and stiffness intensity reported via the PROs
**[52]**	" to compare different data aggregation approaches and machine learning models for the prospective prediction of fall risk using gait parameters derived either from continuous real-world recordings or from unsupervised gait tests."	Case series	Parkinson Disease	35	35			Mobile Gait Lab	tri-axial accelerometer, tri-axial gyroscope	2	foot	Real-world gait, prospective fallers"	against gold standars	Gait parameters, Fall		7	Accuracy/ Reliability	"Our findings suggest that fall risk can be predicted best by merging the entire two-week real-world gait data of a patient, outperforming the prediction using unsupervised gait tests (68.0% balanced accuracy) and contribute to an improved understandin	Accuracy in fall risk prediction: 68%	

																	g of fall risk prediction."		
**[34]**	"To address movement assessments of PD patients with a pair of smart textile gloves"	Case series	Parkinson Disease	4	4		iTex Gloves	tri-axial accelerometer	6	fingers	"system's performance and usability"	against in-clinic assessment		Wearing time, kinematic data	91	Acceptability and Accuracy/ Reliability	"The IoT infrastructure demonstrated robust performance in home settings and offered minimum barriers for the assessment exams and the data communication with a remote server. In the post-study survey, all four participants expressed that the system was easy to use and poses a minimum barrier to performing the test independently."	Acceptability: the system was easy to use and poses a minimum barrier to performing the test independently. Accuracy: Robust performance in home settings and minimum barriers for the assessment exams.	

**[46]**	"To detect missteps under real-world to enhance the evaluation of fall risk"	Case series	Parkinson Disease	33	40	7	DynaPort Hybrid	tri-axial accelerometer, tri-axial gyroscope	1	lower back	"Misstep detection"	against in-clinic assessment	Fall	acceleration parameters from IMU	2	Accuracy/ Reliability	"When we applied this algorithm to the 3 days recordings, patients who reported two falls or more in the 6 months prior to the study (i.e., fallers) were significantly more likely to have a detected misstep during the 3 day recordings (p = 0.010) compared to the non-fallers."	Accuracy: patients who reported two falls or more in the 6 months prior to the study had significantly more likely to have a detected misstep during the 3 day recordings (p = 0.010)
**[45]**	"to identify whether wearable sensor data can be used to objectively quantify symptom severity in individuals with PD exhibiting motor fluctuations."	Case series	Parkinson Disease	28	31	3	GeneActiv	tri-axial accelerometer	3	wrists, waist	"to assess the accuracy of algorithms, to assess feasibility of using such measures outside of the laboratory and compare the outcomes of both data collection paradigms."	against in-clinic assessment	Symptom	clinical and kinematic scores for tremor, dyskinesia, and bradykinesia detected at home compared to motor tasks performed during the laboratory visits	6	Accuracy/ Reliability	"The data challenge showed that this dataset has great potential for enabling important advances in the management of symptoms and motor fluctuations in PD."	Accuracy: great potential of the system

**[47]**	"to develop and evaluate an algorithm for the automated detection of standardized gait tests from real-world IMU data"	Case series	Parkinson Disease	12	12		Mobile Gait Lab	tri-axial accelerometer, tri-axial gyroscope	2	foot	"fully automated processing of IMU recordings including unsupervised daily-living activities and standardized gait tests"	against in-clinic assessment	Quantity of movement, Score/index/test correlation	gait detection compared with 4×10 MWT (meters walking test)	2	Accuracy/ Reliability	"A concurrent validity evaluation revealed very good agreement between spatio-temporal gait parameters derived from manually labelled and automatically detected 4×10 MWTs."	Accuracy: very good agreement between spatio-temporal gait parameters derived from manually labelled and automatically detected 4×10 MWTs."
**[49]**	"to explore the feasibility of a newly developed Parkinson's disease monitoring system, which aims to measure Parkinson's disease symptoms during daily life"	Case series	Parkinson Disease	20	20		MOX 5	tri-axial accelerometer, tri-axial gyroscope	3	wrists, chest	Comfort and reliability	against in-clinic assessment			2	Compliance and Accuracy/ Reliability	"participants wore the wearable sensors during 94% of the instructed timeframe and even beyond. Our Parkinson's disease monitoring system is a feasible method to use in a diverse Parkinson's disease population for at least a period of two weeks. "	Compliance: 94%, Reliability: A preliminary analysis showed that sensor data could reliably predict subjectively reported OFF moments

**[51]**	"to measure FoG during daily life with body-worn sensors"	cohort study	Parkinson Disease	23	48		25	Parkinson Disease	Opal	tri-axial accelerometer, tri-axial gyroscope, tri-axial magnetometer	5	feet, shanks, lower back	"numbers of FoG episodes, percentage of time spent freezing and its variability"	against in-clinic assessment	Symptom	Period of walking (3D angular velocity and 3D acceleration of the lumbar sensor)	730	Accuracy/ Reliability	"percent time spent freezing and the variability of time spent freezing differentiated between people with and without FoG ($p < 0.05$), and that short FoG episodes account for 69% of the total FoG episodes"	Reliability: moderate to good agreement in the number of FoG episodes detected between clinical raters and the algorithm. Average ICC (0.62)	Short FoG episodes account for 69% of the total FoG episodes. Difference in percent time spent freezing and the variability of time spent freezing between people with and without FoG ($p < 0.05$).
**[48]**	"to assess if wearable sensor data can be used to generate accurate estimates of limb-specific symptom severity"	Case series	Parkinson Disease	17	19	2			Shimmer	tri-axial accelerometer	5	forearms, shanks, lower back	"symptom variability observed during medication cycles: tremor, dyskinesia and bradykinesia"	against in-clinic assessment	Symptom	comparison between symptoms and data recorded	912	Accuracy/ Reliability	not available		

**[50]**	"To analyse the responsivity of wearable inertial sensor and to study the ability of the sensor in the detection of MF, dyskinesia, FoG and the percentage of Off-Time, under ambulatory conditions of use."	cohort study	Parkinson Disease	29	39	5	5	Parkinson Disease	STAT-ON	tri-axial accelerometer, tri-axial gyroscope	1	hip	"The mean percentage of Off-Time, the mean percentage of On-Time, the number of steps, motor fluctuation, dyskinesia, FoG and minutes walking per day"	against in-clinic assessment	Symptom, quantity of movement	FOG: Clinical nterview; Motor fluctuation: UPDRS-IV (item 39); Dyskinesia: item 35 of UPDRS-IV	7	n.a.	"The mean percentage of Off-Time among the patients who decreased their Off-Time (79% of patients) was -7.54 ± 5.26. The mean percentage of On-Time among the patients that increased their On-Time (59% of patients) was 8.9 ± 6.46. The Spearman correlation between the mean fluidity of the stride and the UPDRS-III-Factor I was 0.6 (p = <0.001). Change in other sensor-based parameters (dyskinesia, FoG and minutes walking per day) was not significant."	Accuracy: kappa agreement analysis between the UPDRS-IV/clinical interview and the sensor was 0.089 for MF, 0.318 for dyskinesia and 0.481 for FoG.	Mean percentage of Off-Time among the patients who decreased their Off-Time (79% of patients): -7.54 ± 5.26%. The mean percentage of On-Time among the patients that increased their On-Time (59% of patients): 8.9 ± 6.46%. The Spearman correlation between the mean fluidity of the stride and the UPDRS-III- Factor I was 0.6 (p = <0.001). Change in dyskinesia, FoG and

																				minutes walking per day: not significant."
**[37]**	"to evaluate the feasibility of use and quality of data collected by the system, and report on the reliability, validity, and sensitivity to change of a set of digital measures derived from the parkinson's Disease Virtual Motor Exam (PD-VME) during a multi-year deployment in the Personalized Parkinson Project	Case series	Parkinson Disease	370	370			Verily Study watch	tri-axial accelerometer, tri-axial gyroscope	1	wrist	"Engagement, rest remor, upper limb bradykinesia, arm swing during gait"	against in-clinic assessment	Quantity of movement, symptom	accelerometer data, digital signal processing for upper extremity bradykinesia and tremor	2	Compliance	"Median wear-time was 21.1h/day, and 59% of per-protocol remote assessments were completed. Analytical validation was established for in-clinic measurements, which showed moderate-to-strong correlations with consensus MDS UPDRS Part III ratings for rest tremor (ρ=0.70), bradykinesia (ρ=−0.62),	Compliance: Median wear-time was 21.1h/day, and 59% of per-protocol remote assessments were completed. Reliability: in-clinic measurements, which showed moderate-to-strong correlations with consensus MDS UPDRS Part III ratings for rest tremor (ρ=0.70), bradykines	

																	and gait (ρ=−0.46). "	ia (ρ=−0.62), and gait (ρ=−0.46)
**[44]**	a novel fuzzy logic FoG detection algorithm exploiting two spatio-temporal gait parameters mostly related with FoG, i.e. SC and SL, joined with other frequency domain features; • real-time tests of the proposed algorithm implemented on a smartphone, used for both sensing and processing functions, in a sample of patients with PD; • the	Case series	Parkinson Disease	44	44		Smartphone, iPhone 5 and 6 + customized App	tri-axial accelerometer, tri-axial gyroscope	1	iliac crest	Gait detention of FoG, sensitivity of the system	against in-clinic assessment, gold-standard	Symptom, Gait parameters	algorthm to detect gait compared with another algorithm	6	Accuracy/ Reliability	the algorithm reached the following scores for sensitivity, specificity, accuracy, and precision: 96.7%, 98.1%, 97.6%, 96.4%, respectively. The ROC curve (Fig. 4a) encloses an Area Under the Curve (AUC) of 0.9892.	96.7% sensitivity, 98.1% specificity, 97.6% accuracy, 96.4% precision, Area Under the Curve (AUC) of 0.9892.

	validation of the FoG monitoring system against clinicians’ observation in the home environment as well as in the laboratory, and the comparison with the Moore-Bachlin (MB) algorithm, which is the most used FoG detection algorithm.																	
**[53]**	To analyze the ability of the REMPARK System to detect ON-OFF fluctuations in a sample of PD patients	Case series	Parki nson Dise ase	33	4 4	11	REM PAR K	tri-axial acceler ometer	1	iliac crest	"Ability to detect ON-OFF fluctuatio ns"	against patient-reported	Sy mp to m, Gai t par am ete rs	algorth m to detect gait and dyskine sia	7	Accuracy/ Reliability	"The average specificity and sensitivity achieved by the system in recognising ON-OFF motor states is 88% and 97% respectively"	88% specificy, 97% sensitivity

**[70]**	"to assess the feasibility of use of wearable sensors, to determine the activity (lying, sitting, standing, walking) of participants, and to survey participants on their experience."	cohort study	Parkinson Disease	36	56	20	Healthy	BioStamp RC	tri-axial accelerometer	5	chest, limbs	"Physical Activity, adherence"	Quantity of movement	time spent lying, sitting and standing	7	Acceptability	"Individuals with Huntington disease spent over 50% of the total time lying down, substantially more than individuals with prodromal Huntington disease (33%, p = 0.003), Parkinson disease (38%, p = 0.01), and controls (34%; p < 0.001). Most (86%) participants were "willing" or "very willing" to wear the sensors again."	Acceptability: Most (86%) participants were "willing" or "very willing" to wear the sensors again	Huntington disease: over 50% of the total time lying down, substantially more than individuals with prodromal Huntington disease (33%, p = 0.003), Parkinson disease (38%, p = 0.01), and controls (34%; p < 0.001)
**[54]**	"to deliver a wearable computing-based solution for independent motor training and assistance."	Case series	Parkinson Disease	9	9			GaitAssist	tri-axial accelerometer, tri-axial gyroscope, tri-axial magnetometer	2	ankles	" 1) Acceptance rates of the system; 2) FoG distributions and FoG episodes	Symptom	ankle position kinematica data, questionnaire acceptability	2	Acceptability	1) GaitAssist received an average of 4 out of 5 as a wearability score; 2) Until the last day of training, there are no such long FoGs detected, and the distribution of the FoG duration overall decreases and becomes	wearability score: 4 out 5	the distribution of the FoG duration decreases day by day

																more compact. Except for one subject, we did not find a clear trend in reduction of the number of FoG episodes.	
**[61]**	"to explore the acceptability of living with a multimodal sensor platform in a naturalistic setting in PD. Two subobjectives are to identify any suggested limitations and to explore the sensors' impact on participant behaviors."	cohort study	Parkinson Disease	12	24	12	Healthy	not specified	tri-axial accelerometer	2	wrists	Acceptability of: Privacy-Preserving Cameras, Wearables, Other Ambient Sensors, Intermittent High-Resolution Video Data Capture, Optimizing Sensor Platform Design, Sensor Controls.		Quantity of movement	interview	28	Acceptability and Accuracy/ Reliability	"This work has found that it is acceptable to use multimodal sensors, including wrist-worn wearables, cameras, and other ambient sensors passively, in free-living patients with PD but that the least acceptable sensor was the wearable device." " Recording high-resolution videos in a home setting for limited periods was considered acceptable."	the system is acceptable

**[55]**	" to investigate which in-home sensors, in which locations, could gather useful data about fall risk."	Case series	Parkinson Disease	5	6	1		not specified	tri-axial accelerometer, tri-axial gyroscope	1	wrist	"1) To observe people at high risk of falling moving freely at home, noting, and recording:<br>- movement patterns (e.g., habitual activities),<br>- behaviours (likely to increase or decrease fall risk),<br>- locations and actions associated with (historic or observed) falls and near-misses<br>2) To observe participants repeatedly demonstrating one habitual activity they associate		Fall	Video compared sensors data	7	Acceptability	"ur unobtrusive sensors were acceptable to participants: they could detect instability during everyday activity at home and potentially guide intervention. Monitoring the route between chair and stairs is likely to give information without invading the privacy of people at high risk of falling"	Acceptable

												with a particularly high fall risk (e.g., descending steps), recording from multiple camera positions.							
**[41]**	"To evaluate whether a smartwatch and smartphone application could measure features of early PD patients"	cohort study	Parkinson Disease	82	132	50	Healthy	Apple watch 4 or 5, iPhone 10 or 11 + App Brain Baseline	tri-axial accelerometer, tri-axial gyroscope, tri-axial magnetometer	2	wrists	"Gait evaluation, Psychomotor function, tremor"	Symptom, quantity of movement	arm swing, stance time, gait cadence, total double support time, turn duration, finger-tapping tasks.	3	n.a.	"Features derived from the devices, particularly arm swing, the proportion of time with tremor, and finger tapping, differed significantly between individuals with early PD and age-matched controls"		Arm swing, the proportion of time with tremor, and finger tapping, differed significantly between individuals with early PD and age-matched controls
**[57]**	"to evaluate the feasibility of a wrist-worn wearable accelerometer system to detect PD	Case series	Parkinson Disease	6	6			Axivity AX3	tri-axial accelerometer	1	wrist	comparison between a new system (CNN-T/NT + MLP) to	Symptom	algorithm to detect presence or absence of tremor	3	Accuracy/ Reliability	" CNN-T/NT + MLP performs better than Zhang et al., with an average error of 4.1% when	average error: 9.1% when predicting percentage of tremor time on WILD data	

	tremor in the wild (uncontrolled scenarios)"											remotely detect tremor and other systems already used		in the accelerometer signals			predicting percentage of tremor time on LAB data, and 9.1% on WILD data."	
**[40]**	"to investigate whether turning in the daily living home environment in subjects with Parkinson Disease who experience FoG is more impaired than in subjects without FoG."	cohort study	Parkinson Disease	257	326	69	Parkinson Disease	DynaPort Hybrid	tri-axial accelerometer, tri-axial gyroscope	1	lower back	"Quantity of turning, quality of turns"	Turning events	Accelerometer data for turn angle amplitude, velocity, number, duration, jerkiness	3	n.a.	"The number of turns (19.3 ± 9.2/30 min in freezers, 22.4 ± 12.9/30 min non-freezers; p = 0.194) was similar in the two groups. Some aspects of quality of turns, specifically mean jerkiness, mean and variability of medio-lateral jerkiness were significantly higher (p < 0.05) in the freezers, compared to non-freezers. Interestingly, subjects with FoG showed specific turning differences in the turns with larger angles compared to those without FoG."	The number of turns (19.3 ± 9.2 /30 min in freezers, 22.4 ± 12.9 /30 min non-freezers; p = 0.194) was similar in the two groups. Mean jerkiness, mean and variability of medio-lateral jerkiness: significantly higher (p < 0.05) in the freezers. Interestingly, subjects with FoG showed specific turning differences in the turns with larger angles compared to those

																				without FoG.
**[67]**	"to test whether objectively assessed physical behavior parameters could con tribute to the discrimination of cognitive subtypes of Parkinson Desease (PD), especially between PD Dementia and PD-Mild cognitive impairment"	cohort study	Parkinson Disease	48	5 5	7		Dyna Port Mini mod	tri-axial accelerometer, tri-axial gyroscope	1	lower back	"Physical Behavior Outcomes (time spent lying, sitting, standing, walking, shuffling and not-wearing sensor)."		Quantity of movement	algorithms activity parameters (amount of total time spent in each behavior, total number of bouts per day for each behavior, the mean vector magnitude of dynamic acceleration per day for	2	n.a.	" PDD patients showed fewer sedentary bouts than non-ADL-impaired PD-MCI and PD-NC patients, as well as a longer sedentary bout length. These differences were mainly caused by fewer but longer sitting bouts."		PDD patients: longer sedentary bout length, fewer sedentary bouts than non-ADL-impaired PD-MCI and PD-NC patients.

														each behavior					
**[62]**	"to objectively quantify daily motor fluctuations and the usefulness of combined EMG and ACC measurements to assess possible changes of the neuromuscular activity in advanced PD patients before and after reprogramming of DBS"	cohort study	Parkinson Disease	9	16	7	Parkinson Disease	Faros EMG	Surface EMG, tri-axial accelerometer	1	arm	Correlation between EMG+ACC paramters and Unified Parkinson's Disease Rating Scale (UPDRS), Comparison between home diaries (filled at home by patients) and detected EMG+ACC data"	Scale/index/test correlation, quantity of movement	Unified Parkinson's Disease Rating Scale Part 3, EMG data	7	n.a.	"The combination of EMG and ACC parameters (first principal component PC1) correlated significantly with patient's condition as quantified by the motor score of Unified Parkinson's Disease Rating Scale and it changed significantly with dDBS reprogramming in line with decreased PD symptoms. Monitoring data detected in comparison with the home diaries: 91 % concordance with tremor, 76 % with rigidity, and	Monitoring data compared to home diaries: 91% tremor, 71% rigidity, 74% dyskinesia	great correlation between EMG and ACC parameters and UPDRS score.

																		74 % with dyskinesia."		
**[56]**	"To evaluate the associations among FOG measured during unsupervised daily-living monitoring, structured in-home FOG-provoking tests, and self-report."	Case series	Parkinson Disease	28	28			Gait Tutor system	tri-axial accelerometer, tri-axial gyroscope	3	shoes, pocket	"associations among FOG measured during unsupervised daily-living monitoring, structured in-home FOG-provoking tests, and self-report."		Symptom	time spent frozen	2	n.a.	The %TF during unsupervised daily living was mild to moderately correlated with the %TF during a subset of the tasks of the in-home off-medication testing but not the on-medication testing or self-report		%TF (time spent frozen) during was mild to moderately correlated with the %TF during the in-home off-medication testing but not the on-medication testing or self-report

**[60]**	"to assess the impact of motion sensor-based telehealth diagnostics on PD clinical care and management"	randomized controlled trial	Parkinson Disease	9	18	9	Parkinson Disease	Kinesia	tri-axial accelerometer	1	finger	"Compliance"		percentage of weeks a participant completed at least 6 Kinesia assessments on at least 1 day during the week; software Kinesia platform	91	Compliance	"Homebased assessments were completed with a median compliance of 95.7%. For a subset of participants, the neurologist successfully used information in the reports, such as quantified responses to treatment or progression over time, to make therapy adjustments. Changes in clinical characteristics from study start to end were not significantly different between the groups."	Compliance: 95,7%	Changes in clinical characteristics from study start to end were not significantly different between the groups

**[58]**	"To evaluate if measures from wearable sensors can be used as future surrogates for the MDS-UPDRS rigidity and Postural Instability and Gait Difficulty (PIGD) subscores"	Case series	Parkinson Disease	31	31		Opal	tri-axial accelerometer, tri-axial gyroscope, tri-axial magnetometer	3	socks, waist	"activity quantity and quality, turning" compared with laboratory evauations about Postual instability and gait difficulty (PIGD)	Turning events, gait parameters, quantity of movement	number of walking bouts, number of turns; gait speed, foot pitch angle at heel strike and variability of both; turn duration, turn velocity, turn angle, and the variability of each	28	Accuracy/ Reliability	"The number of walking bouts and turns correlated significantly with the rigidity subscore, while the number of turns, foot pitch angle, and sway area while standing correlated significantly with the Postural Instability and Gait Difficulty (PIGD) subscore ($p<0.05$). The multivariable linear regression showed that rigidity subscore was best predicted by the number of walking bouts while the PIGD subscore was best predicted by a combination of number of walking bouts, gait speed, and postural sway."	Accuracy: The multivariable linear regression showed that rigidity subscore was best predicted by the number of walking bouts while the PIGD subscore was best predicted by a combination of number of walking bouts, gait speed, and postural sway.	number of walking bouts and turns: significantly correlated with the rigidity subscore. The number of turns, foot pitch angle, and sway area while standing: significantly correlated with the Postural Instability and Gait Difficulty (PIGD) subscore ($p<0.05$)

**[63]**	"To show that continuous monitoring of natural turning with wearable sensors during daily activities inside and outside the home is feasible for people with PD and elderly people"	cohort study	Parkinson Disease	12	30	18	Healthy	Opal	tri-axial accelerometer, tri-axial gyroscope, tri-axial magnetometer	3	belt, feet	"1) to detect and analyze prescribed and spontaneous turns during gait in the laboratory and home; 2) to demonstrate the validity of our inertial algorithm in both the laboratory and home environment"	Turning events	algorithm estimating turn events (duration, peak and mean velocity, number of steps to complete a turn and body jerk during a turn).	210	Accuracy/ Reliability	" the algorithm maintained a sensitivity of 0.90 and 0.76 and a specificity of 0.75 and 0.65, respectively. Second, we apply the turning algorithm to data collected in the home from 12 PD and 18 CT subjects. The algorithm successfully detects turn characteristics, and the results show that, compared to controls, PD subjects tend to take shorter turns with smaller turn angles and more steps. Furthermore, PD subjects show more variability in all turn metrics throughout the day and the week."	Sensitivity 90-76%, Specificity 75-65%	PD subjects tend to take shorter turns with smaller turn angles and more steps, more variability in all turn metrics.

**[66]**	"to discriminate fallers from non-fallers with PD in the clinic and during daily life."	cohort study	Parkinson Disease	17	34	17	Parkinson Disease	Opal	tri-axial accelerometer, tri-axial gyroscope, tri-axial magnetometer	3	feet, lower back,	Gait and turning (in fallers and non-fallers, in ON and OFF levodopa state)	Turning events, gait parameters, quantity of movement	Mean and variability of strides, turn: rotation around the vertical plane with a minimum of 40°/s, and a start and end of the turn was defined with a threshold of 15°/s."	5	n.a.	"digital measures collected in the off levodopa state were significantly different between groups, (average turn velocity, average number of steps to complete a turn, and variability of gait speed, P < 0.03). During daily life, the variability of average turn velocity (P = 0.023) was significantly different in fallers than non-fallers."	Positive falls history: not significantly associated with turning parameters
**[68]**	"to study diurnal patterns of motor performance in a large cross-sectional cohort of de novo PD patients. In addition, we aimed to corroborate these findings in a longitudinal setting using objective	cohort study	Parkinson Disease	12	12			Parkinson's Kinetigraph™	tri-axial accelerometer	1	wrist	Diurnal variaions of Bradykinesia	Symptom	algotithm (median value of bradykinesia over a specific period during the day)	1	n.a.	"diurnal variations were present in percentage of time spent in severe bradykinesia (p < 0.001) with the lowest percentage during the 09:00-12:00 epoch (69.56±16.68 %), when most patients are awake and start daily activity,	percentage of time spent in severe bradykinesia (p < 0.001): the lowest percentage during the 09:00-12:00 epoch (69.56±16.68%), the highest percentage during the 18:00-

	wearable sensor data."																	and the highest percentage during the 18:00-21:00 epoch (73.58±16.35%)."		21:00 epoch (73.58±16.35%)
**[105]**	"To determine whether a multimodal telemedical intervention is feasible in patients with PD and to identify barriers for handling and usability of its components"	Case series	Parkinson Disease	11	12	1			PDmonitor	Tri-axial accelerometer, tri-axial gyroscope	5	arms, legs, trunk	Patients's adherence		% of valid measures	7	Compliance	"The wearable sensors recorded 71% of valid daily measurements."	Compliance: 71% of vaid daily measurement	
**[28]**	"to quantify the hazard ratio of falls in PD patients in real life."	cohort study	Parkinson Disease	2063	4126		2063	Fallers without Parkinson Disease	PERS	tri-axial accelerometer, barometer	1	neck	"incidence rate of any fall, incidence rate of a new fall after enrollment, 1-year cumulative incidence of falling."	Fall	algorithm including automatic detected falls	14	n.a.	"The incidence rate of any fall was higher among self-reported PD patients than controls (2.1 vs. 0.7 falls/person, respectively; P < .0001). The incidence rate of a new fall after enrollment (ie, hazard ratio) was 1.8 times higher for self-reported PD		Incidence rate of any fall: higher among self-reported PD patients than controls (2.1 vs. 0.7 falls/person, respectively; P < .0001). The incidence rate of a new fall after

																	patients than controls (95% confidence interval, 1.6–2.0)."		enrollment (ie, hazard ratio): 1.8 times higher for self-reported PD patients than controls (95% confidence interval, 1.6–2.0
**[64]**	"to evaluate the effect of fear of falling (FOF) on the mobility of 26 Prkinson's desease"	cohort study	Parkinson Disease	9	26	17	Parkinson Disease	Reha Gait	tri-axial accelerometer	1	lower back	1) mobility differences between patients with PD with or without FOF. 2) to determine whether daily activity assessment can complement lab assessment. 3) studying differences in PD-FOF+ and PD-FOF− parameters.	Quantity of movement, gait parameters	gait speed, sit-to-stand peak power, and peak angular velocity compared between lab and daily activities.	12	Accuracy/ Reliability	" The peak angular velocity of the turn-to-sit transition of the timed-up-and-go (TUG) test had the highest discriminative power between PD-FOF+ and PD-FOF− (r-value of effect size = 0.61). Moreover, PD-FOF+ had a tendency toward lower gait speed at home and a lower amount of walking bouts, especially for shorter walking bouts."	Accuracy: The combination of lab and daily activity parameters reached a higher discriminative power [area under the curve (AUC) = 0.75]	PD-FOF+: lower gait speed at home, lower amount of walking bouts. peak angular velocity of the turn-to-sit transition of the timed-up-and-go (TUG) test had the highest discriminative power between PD-FOF+ and PD-FOF−

**[65]**	"To demonstrate if Fear of Falling (FOF) and a history of falls, independentl y affect turning metrics in both (laboratory and home) environments ."	cohort study	Parki nson Dise ase	40	4 0			Reha Gait	tri-axial acceler ometer	1	lowe r back	Number of turning, maximu m and average angula velocity on turning	Tur nin g eve nts	Turn: duratio n, angle, average angular velocity , starting angular velocity , middle angular velocity , ending angular velocity and maxim um angular velocity of every turn.	7	n.a.	"In the home evaluations, a lack of FOF was associated with lowered maximum and average angular velocities of turns. Positive falls history was not significantly associated with turning parameters, neither in the lab nor in the home."	lack of FOF was associate d with lowered maximu m and average angular velocities of turns. Positive falls history was not significan tly associate d with turning paramete rs, neither in the lab nor in the home
**[69]**	"to evaluate if balance and timed-up-and-go data obtained by a smartphone application during COVID-19 lockdown were able to predict falls in PD patients."	cohort study	Parki nson disea se	29	3 3	4	Parki nson Disea se	Smar tpho ne App Ence phaL og Hom eTM	tri-axial acceler ometer, tri-axial gyrosco pe	1	not speci fied	"stand-up time, magnitud e of mediolate ral sway at the 3mTUG, static balance."	Qu ant ity of mo ve me nt, Sy mp to m	Mean kinema tic values	3	n.a.	"The “fallers” showed a longer stand-up time and a higher magnitude of mediolateral sway at the 3mTUG than the “non-fallers” patients. There were no differences across groups in the other 3mTUG parameters and in the static balance examination"	fallers: longer stand-up time and a higher magnitud e of mediolate ral sway at the 3mTUG. There were no difference s across groups in the other 3mTUG paramete rs and in the static balance

																			examination
**[59]**	"To test the feasibility and potential efficacy of a wearable sensor-enabled technological platform designed for an in-home music-contingent stepping-in-place (SIP) training program to improve step automaticity during dual-tasking"	non-randomized controlled trial	Parkinson Disease	6	11	5	Parkinson Disease	Smartphone, iPod touch (4th generation), Gait Reminder™ App	tri-axial accelerometer, tri-axial gyroscope	1	knee	Fear of falling (FES-I), and Dual task (DT) step automaticity were evaluated.	Fall	Dual task cognitive score compared with FES-I, MoCA, FOG-Q questionnaire.	7	n.a.	"While we found no significant effect of training on FES-I, MoCA, or FOG-Q, we did observe a significant group (music vs podcast) by training interaction in DT step automaticity".		No significant effect on FES-I, MoCA, or FOG-Q. Significant group (music vs podcast) by training interaction in Dual Task step automaticity".
**[38]**	"to assess the feasibility of implementing wearable technology to collect data from multiple sensors during the daily lives of PD patients"	Case series	Parkinson Disease	805	805			SmartPhones, Pebble Smartwatch (App name "Fox wearable companion")	tri-axial accelerometer, tri-axial gyroscope	2	wrist, pocket	Compliance, system usability		Hours/participant/day, system usability scale	28	Compliance	"Participants were compliant for 68% (16.3 hours/participant/day) of the study period in NL and for 62% (14.8 hours/participant/day) in NAM." "The system usability scale median score was 62.5 (1st and 3rd QT 47.5–	Compliance: 68% (16.3 hours/participant/day) in NL, 62% (14.8 hours/participant/day) in NAM. Usability scale median score: 62.5, category between “Ok” and “Good”	

															72.5), which classifies the wearable platform in a category between “Ok” and “Good”	
**[39]**	"to investigate whether the severity of motor fluctuations is associated with changes in physical activity patterns in a large cohort of People with Parkinson."	Case series	Parkinson Disease	304	304	SmartPhones, Pebble Smartwatch (App name "Fox wearable companion")	tri-axial accelerometer, tri-axial gyroscope	2	wrist, pocket	" Association between severity of motor fluctuations and mean time spent walking per day, Association between severity of motor fluctuations and mean time spent walking per day after levodopa intake."	Symptom, quantiy of movement	mean time spent walking in the second hour after levodopa intake (post-levodopa activity) and the mean time spent walking in the last hour before levodopa intake (pre-levodopa activity) per individual	3	n.a.	"The severity of motor fluctuations did not influence the mean time spent walking (B=2.4±1.9, p=0.20), but higher age (B=-1.3±0.3, p=<0.001) and greater severity of motor symptoms (B=-0.6±0.2, p<0.001) was associated with less time spent walking (F(3,216) = 14.6, p<.001, R2 =.17). The severity of fluctuations was not associated with the amount of change in time spent walking in relation to	Mean time spent walking: Not influenced by severity of motor fluctuations. Less time spent walking: associated to higher age and greater severity of motor symptoms. Severity of fluctuations not associated with the amount of change in time spent walking in

																	levodopa intake in any part of the day. "		relation to levodopa intake in any part of the day.
**[98]**	"s to discuss implementation of the telemedicine technology for rehabilitation of patients with injuries of the lower extremities"	non-randomized controlled trial	Polytrauma	32	48	16	Polytrauma	not specified	tri-axial accelerometer	1	knee	"time to consult patients, Patient satisfaction"		Minutes	21	Acceptability	"The orthopedic surgeon during telerehabilitation took significantly less time to consult patients (1.9 minutes) than the traditional rehabilitation (15.2 minutes). Patient satisfaction was higher for the telerehabilitation with machine learning algorithm (78.3%) than for the orthopedic surgeon's traditional	Satisfaction: machine learning algorithm (78.3%), orthopedic surgeon's traditional rehabilitation (36.7%)	less time to consult patients (1.9 minutes) than the traditional rehabilitation (15.2 minutes)

																	rehabilitation (36.7%)"	
**[62]**	To develop and test wearable monitoring of surface electromyography and motion for detection and quantification of positive and negative myoclonus in patients with progressive myoclonic epilepsy type 1 (EPM1).	Case series	Progressive myoclonic epilepsy type 1	23	2 3		Faros EMG	Surface EMG, tri-axial accelerometer	2	biceps brachii, extensor digitorum	detection of myoclonus; the association between the calculated surface EMG and ACC parameters with myoclonus severity of arms	against in-clinic assessment, against patent-reported	Symptom, quantity of movement, scale/index correlation	EMG and ACC data	2	Accuracy/ Reliability	Correlation analysis showed moderate to strong correlations between the UMRS-based myoclonus frequency × amplitude scores and the EMG and acceleration parameters; The monitoring-based MI correlates strongly and significantly with the UMRS-based frequency × amplitude in both arms (ϱ = 0.883 for dominant BB and ϱ = 0.823 for non-dominant EDC, p < 0.001).; EMG- and ACC-based	Reliability: The monitoring-based MI correlates strongly and significantly with the UMRS-based frequency × amplitude in both arms (ϱ = 0.883 for dominant BB and ϱ = 0.823 for non-dominant EDC, p < 0.001).; EMG- and ACC-based parameters followed the subjective diary-based changes in

																	parameters followed the subjective diary-based changes in MD during the 48-hour long monitoring. (reported in one case)	MD during the 48-hour long monitoring.	
**[103]**	"to investigate the single-day reliability of various Physical Activity (PA) measures in patients with a Spinal Cord Injury (CSI) and to propose recommendations on how many days of PA measurements are required to obtain reliable results."	Case series	Spinal cord injury	63	63		ReSense	tri-axial accelerometer, tri-axial gyroscope, tri-axial magnetometer, barometer	3	wrists, wheel	"total activity counts, PA intensity levels, metrics of wheeling quantity and metrics of movement quality."	against in-clinic assessment	Quantity of movement	IMU data (the total distance and the distance wheeled actively, upper limb movement laterality and mean velocity) reliability	3	Accuracy/ Reliability	"Activity counts showed consistently high single-day reliabilities, while measures of PA intensity levels considerably varied depending on the rehabilitation progress. Single-day reliabilities of metrics of movement quantity decreased with rehabilitation progress, while metrics of movement	Reliability: consistently high single-day reliabilities. Single-day reliabilities of metrics of movement quantity decreased with rehabilitation progress, while metrics of movement quality increased	PA intensity levels considerably varied depending on the rehabilitation progress

																	quality increased. "		
**[71]**	"to determine adherence with wearing a consumer-grade step activity monitor, to determine accuracy of step counts compared with a manual tally; calculate correlations between the Fitbit step counts and the mobility performance scores at discharge and 30 days after stroke; determine variability and change in weekly step counts over 90 days; and evaluate patient experience with using the Fitbit	Case series	Strok e	15	1 5		Fitbit Char ge	tri-axial acceler ometer	1	wrist	"fitbit accuracy, variabilit y step-per-day, mobility performa nce."	against in-clinic assessme nt	Qa unt iy of mo ve me nt	Variabi lity of step per day	3	Accuracy/ Reliability	"Mean daily step count in the first week after discharge was 4376. Daily step counts increased slightly during the first 30 days after discharge (average increase of 52.5 steps/day; 95% CI 32.2-71.8) and remained stable during the 30-90 day period after discharge. Mean step count difference between step activity monitor and manual tally was –4.8 steps (–1.8%). Intraclass correlation	Intraclass correlation coefficient s for step counts and 2-minute walk, standing balance, and 4-meter gait speed at discharge: 0.41 (95% CI –0.14 to 0.75), –0.12 (95% CI –0.67 to 0.64), and 0.17 (95% CI –0.46 to 0.66), respectivel y.	Mean daily step count in the first week after discharge : 4376. Daily step counts increased slightly during the first 30 days after discharge (average increase of 52.5 steps/day ; 95% CI 32.2-71.8). Mean step count difference between step activity monitor and manual tally: 4.8

	Charge HR poststroke."																	coefficients for step counts and 2-minute walk, standing balance, and 4-meter gait speed at discharge were 0.41 (95% CI –0.14 to 0.75), –0.12 (95% CI –0.67 to 0.64), and 0.17 (95% CI –0.46 to 0.66), respectively. "		steps (–1.8%).
**[72]**	"to quantify arm use in hemiparetic patients."	cohort study	Stroke	5	10	5	Healthy	not specified	tri-axial accelerometer	2	wrists	"mean arm use, simmetry in arm use"	against in-clinic assessment	Quantity of movement	Video compared sensors data	56	Accuracy/ Reliability	"The total arm use (area under ϱ(ϕ)) for patients is lower than that of healthy controls, three patients presented a bias towards using the less-affected arm."	Average accuracy: 47%	The total arm use (area under ϱ(ϕ)) for patients is lower than that of healthy controls.

**[75]**	"To evaluate the feasibility and preliminary effects of a sedentary behavior change intervention on sedentary behavior, physical activity, function, and quality of life following inpatient stroke rehabilitation."	Case series	Stroke	32	34	2		ActivPAL	tri-axial accelerometer	1	wrist	"reach, retention, participants' satisfaction with the program, compliance with wearing the activity monitors, sedentary behavior, physical activity, impairment, mobility, cognitive status, quality of life, and self-efficacy.		Quantity of movement		90	Acceptability	"Satisfaction with the program was 89%. Sedentary time decreased by 54.2±13.7 minutes per day (P<.01) at postintervention and 26.8±14.0 minutes per day (PZ.07) at follow-up, relative to baseline. There were significant improvements in walking speed, cognition, impairment, and self-reported quality of life over time (P<.05). Self-efficacy was high across all time points. The number of steps and time spent stepping were not statistically different across both time periods."	Satisfaction: 89%	Sedentary time: decreased by 54.2±13.7 minutes per day (P<.01) at postintervention and 26.8±14.0 minutes per day (PZ.07). iWalking speed, cognition, impairment, and self-reported quality of life over time: Significant improvements (P<.05). Self-efficacy was high across all time points. The number of steps and time spent stepping: not statistically different across both time periods

**[74]**	"to compare the functional ability and daily use of the affected Upper Extremity (UE) of individuals with stroke between discharge to home and 12 months after stroke, to predict the UE daily use 12 months after stroke"	Case series	Stroke	32	58	26			Actical	tri-axial accelerometer	1	wrist	"UE functional ability improvement from discharge to 12 months after stroke, daily use of the nonaffected UE	Quantity of movement	algorithm to determine predictors that explain e upper extremity daily use at 12 months after stroke	420	n.a.	"The UE functional ability improved significantly from discharge to 12 months after stroke, the daily use of the nonaffected UE was 3 times more than the affected UE."		"UE functional ability: significantly improved from discharge to 12 months after stroke. Daily use of the nonaffected UE: 3 times more than the affected UE.
**[76]**	"to explore the usability of a wristwatch device called "Smart reminder" for home-based upper limb telerehabilitation for persons with stroke."	Case series	Stroke	11	11				not specified	tri-axial accelerometer, tri-axial gyroscope	1	wrist	"compliance and acceptability"	Quantity of movement	Number of completed exercise sessions	14	Compliance	"high therapy compliance rate (mean = 91%)."	Compliance rate: 91%	
**[73]**	to demonstrate that the supported in-home rehabilitation system has the potential to reduce the cost and the effort for patients and doctors while still maintaining the quality	randomized controlled trial	Stroke	6	12		6	Stroke	not specified	tri-axial accelerometer	2	wrists, elbow	Increase in Motricity Index and Brunnstrom Stage	Scale/index/test correlation, quantity of move	algorithm to detect kinematic data	7	Accuracy/ Reliability	The results demonstrated that the patients from the experimental group had experienced a steady increase in MI throughout the program. They also had improved BS	Accuracy: well correlated, Feasibility: yes	Increase in MI and BS

and effectiveness of rehabilitation	me nt	by at least one stage, which outperforme d the control group in average

Part 3 of 3: Data extraction on systems certification and ethical issues in the studies

Reference number	CE validation	Type of commercialization	Validation	Coder	ethics	conflict disclosure	conflicts presence	Coder ethics & conflicts
**[102]**	yes	commercial device	yes	AP	yes	disclosed	no	NB
**[94]**	yes	medical device	yes	AP	yes	disclosed	yes	NB
**[84]**	yes	medical device	yes	AP	yes	disclosed	no	NB
**[33]**	yes	medical device	yes	AP	yes	disclosed	no	NB
**[88]**	no	customized device	no	NB	no	not disclosed	no	NB
**[87]**	yes	medical device	yes	AP	yes	disclosed	no	NB
**[85]**	yes	medical device	yes	AP	yes	disclosed	no	NB
**[86]**	yes	commercial device	yes	AP	yes	not disclosed	no	NB
**[95]**	yes	commercial device	yes	AP	yes	disclosed	no	NB
**[96]**	yes	commercial device	yes	AP	yes	disclosed	no	NB
**[100]**	yes	commercial device	yes	AP	yes	disclosed	yes	NB
**[93]**	yes	commercial device	yes	AP	yes	disclosed	yes	NB
**[91]**	yes	medical device	yes	AP	yes	disclosed	yes	NB
**[97]**	no	commercial device	yes	AP	yes	disclosed	no	NB
**[99]**	yes	medical device	yes	AP	yes	disclosed	no	NB
**[80]**	yes	medical device	yes	AP	yes	disclosed	yes	NB
**[82]**	yes	medical device	yes	AP	yes	disclosed	yes	NB
**[81]**	yes	medical device	yes	AP	yes	disclosed	yes	NB
**[42]**	yes	medical device	yes	AP	yes	disclosed	yes	NB
**[83]**	N.a.	N.a.	no	GF	yes	disclosed	no	NB
**[43]**	yes	commercial device	yes	AP	yes	disclosed	yes	NB
**[78]**	no	commercial device	no	AP	yes	disclosed	no	NB
**[77]**	yes	commercial device	yes	AP	yes	disclosed	yes	NB
**[79]**	yes	commercial device	yes	AP	yes	disclosed	yes	NB
**[104]**	yes	medical device	yes	AP	yes ("ETHICAL PUBLICATION STATEMENT")	disclosed		GF
**[92]**	no	customized device	yes	AP	no	disclosed		GF
**[89]**	yes	medical device	yes	AP	yes	disclosed		GF

**[90]**	yes	medical device	yes	AP	yes	disclosed		GF
**[52]**	no	Dispositivo customized	no	AP	yes	not disclosed		GF
**[34]**	no	dispositivo customized	no	AP	no	disclosed		GF
**[46]**	yes	medical device	yes	AP	no	disclosed		GF
**[45]**	yes	dispositivo commerciale	no	AP	no	disclosed		GF
**[47]**	no	Dispositivo customized	no	AP	yes	not disclosed		GF
**[49]**	yes	commercial device	yes	AP	yes	disclosed		GF
**[51]**	yes	commercial device	yes	AP	yes	disclosed	yes	GF
**[48]**	yes	medical device	yes	AP	yes	disclosed	no	AP
**[50]**	yes	commercial device	yes	AP	yes	disclosed	no	AP
**[37]**	yes	commercial device	yes	AP	yes	disclosed	yes	AP
**[44]**	yes	medical device	yes	AP	yes	disclosed	no	AP
**[53]**	yes	medical device	yes	AP	yes	disclosed	yes	AP
**[70]**	yes	medical device	yes	AP	yes	disclosed		GF
**[54]**	no	customized device	no	AP	no	not disclosed		GF
**[61]**	yes	commercial device	yes	AP	yes	disclosed		GF
**[55]**	yes	commercial device	yes	AP	yes	disclosed		GF
**[41]**	yes	medical device	yes	AP	yes	disclosed	yes	GF
**[57]**	no	customized device	no	AP	no	disclosed		GF
**[40]**	yes	medical device	yes	AP	no	disclosed		GF
**[67]**	yes	commercial device	yes	AP	yes	disclosed	yes	GF
**[62]**	N.a.	N.a.	N.a.	AP	no	disclosed	yes	GF
**[56]**	no	customized device	yes	AP	yes	disclosed		GF
**[60]**	no	Dispositivo customized	no	AP	yes	disclosed	yes	GF
**[58]**	yes	commercial device	yes	AP	yes	disclosed	yes	GF
**[63]**	yes	commercial device	yes	AP	no	disclosed	yes	GF
**[66]**	yes	commercial device	yes	AP	yes	disclosed	no	AP
**[68]**	yes	commercial device	yes	AP	yes	disclosed	yes	AP

**[105]**	yes	medical device	yes	AP	yes	disclosed	no	AP
**[28]**	yes	medical device	yes	GF	no	disclosed	yes	AP
**[64]**	yes	medical device	yes	AP	yes	disclosed	no	AP
**[65]**	yes	medical device	yes	AP	yes	disclosed	yes	AP
**[69]**	yes	medical device	yes	AP	yes	disclosed	no	AP
**[59]**	yes	medical device	yes	AP	yes	disclosed	no	AP
**[38]**	yes	medical device	yes	AP	yes	disclosed	yes	AP
**[39]**	no	customized device	no	AP	yes	disclosed	yes	AP
**[98]**	no	customized device	no	AP	yes	disclosed	no	AP
**[62]**	n.a,	n.a.	N.a.	AP	yes	disclosed	yes	AP
**[103]**	no	medical device	yes	AP	yes	disclosed	no	AP
**[71]**	yes	medical device	yes	AP	yes	disclosed	no	AP
**[72]**	no	commercial device	yes	AP	yes	disclosed	yes	AP
**[75]**	yes	medical device	yes	AP	yes	not disclosed		AP
**[74]**	yes	medical device	no	AP	yes	not disclosed		AP
**[76]**	yes	medical device	yes	AP	yes	disclosed	yes	AP
**[73]**	no	customized device	no	AP	yes	not disclosed		AP
