# Supplementary material for: Continuous Movement Monitoring at Home Through Wearable Devices: A Systematic Review
Source: Sensors (Basel). 2025 Aug 8;25(16):4889. doi: 10.3390/s25164889 (PMC12389529; doi:10.3390/s25164889)
Supplement: Supplementary file 1 [file sensors-25-04889-s001.zip › Appendix S3.pdf]

Appendix S3. Quality appraisal of the included studies.

	Eligibility Criteria	Whether Participants Formed a Consecutive, Random, or Convenience Series	Rationale for Choosing the Reference Standard (If Alternatives Exist)	Definition of and Rationale for Test Positivity Cut-Offs or Result Categories of the Index Test, Distinguishing Pre-specified from Exploratory	Methods for Estimating or Comparing Measures of Diagnostic Accuracy	How Missing Data on the Index Test and Reference Standard Were Handled	Intended Sample Size and How It Was Determined	Flow of Participants, Using a Diagram	Baseline Demographic and Clinical Characteristics of Participants	Distribution of Severity of Disease in Those with the Target Condition	Estimates of Diagnostic Accuracy and Their Precision (Such as 95% Confidence Intervals)	Any Adverse Events from Performing the Index Test or Using the Reference Standard
[94]	+	-	+	+	+	?	-	-	+	+	+	-
[102]	+	-	+	+	?	?	-	-	+	+	+	-
[88]	+	-	+	+	+	-	-	-	+	+	+	-
[87]	+	+	+	?	?	?	-	-	+	+	+	-
[85]	-	-	-	-	+	?	-	-	+	+	+	-
[84]	+	-	-	-	+	?	-	-	-	-	+	-
[86]	+	-	+	?	?	-	-	-	+	+	+	-
[33]	+	-	-	-	+	?	-	-	+	+	-	-

[95]	+	-	+	+	+	?	-	-	+	+	+	-
[96]	+	-	+	+	+	?	-	-	+	+	+	-
[100]	-	-	+	+	+	?	-	-	+	+	+	-
[93]	+	-	+	+	+	+	+	-	+	+	+	-
[91]	+	-	+	+	+	?	-	-	+	+	+	-
[97]	+	-	-	?	?	?	-	-	-	-	+	-
[99]	+	-	+	+	+	?	-	-	+	+	+	-
[82]	+	-	+	+	+	?	-	-	-	+	+	-
[80]	+	-	+	-	+	?	-	-	+	+	+	-
[81]	+	-	+	?	?	?	-	-	+	+	+	-
[42]	+	-	+	-	+	-	-	-	+	+	+	-
[83]	+	-	-	-	+	?	-	-	+	+	-	-

[78]	+	-	+	-	+	?	-	-	+	+	+	-
[77]	+	-	-	+	+	?	-	-	+	+	+	-
[43]	+	-	+	+	+	-	-	-	+	+	+	-
[79]	+	-	+	+	+	?	-	-	+	+	+	-
[104]	+	-	+	?	?	-	-	-	-	-	+	-
[92]	+	-	+	-	+	?	-	-	+	+	+	-
[89]	+	+	+	?	?	?	-	-	+	+	+	+
[90]	?	-	+	+	+	?	-	+	+	+	+	-
[41]	+	-	+	+	+	?	-	-	+	+	+	-
[46]	+	-	+	+	+	-	-	-	+	+	+	-
[40]	+	-	+	?	+	?	-	-	+	+	+	-
[67]	+	-	+	+	+	-	-	-	+	+	+	-

[62]	+	-	+	+	+	?	-	-	+	+	+	-
[56]	+	-	+	+	+	?	-	-	+	+	+	-
[54]	+	-	+	?	+	?	-	-	+	+	-	-
[45]	+	-	+	+	+	-	-	+	+	+	+	-
[34]	+	-	+	+	+	?	-	-	-	-	+	-
[60]	+	+	+	+	+	?	-	-	+	+	+	-
[52]	+	-	+	+	+	?	-	-	+	+	+	-
[47]	+	-	+	+	+	?	-	-	+	+	+	-
[49]	+	-	+	+	+	?	-	-	+	+	+	-
[61]	-	+	?	?	+	?	-	+	+	+	+	-
[55]	-	-	+	-	+	-	-	-	+	+	-	-
[51]	+	-	+	+	+	?	-	-	+	+	+	-

[58]	+	-	+	+	+	?	-	-	+	+	+	-
[63]	+	-	+	-	+	?	-	-	-	-	+	-
[66]	+	-	+	?	+	?	-	-	+	+	+	-
[68]	+	-	+	-	+	?	-	-	+	+	+	-
[105]	+	-	+	?	+	-	-	-	+	+	+	-
[28]	+	+	+	?	+	?	-	-	+	+	+	-
[64]	+	-	+	+	+	?	-	-	+	+	+	-
[65]	+	-	+	+	+	?	-	-	+	+	+	-
[53]	+	-	+	+	+	-	-	+	+	+	+	-
[48]	+	-	+	-	+	+	-	+	-	-	-	-
[69]	+	-	+	-	+	?	-	-	+	+	+	-
[44]	+	-	+	+	+	?	-	-	+	+	+	-

[59]	+	+	+	?	?	?	-	-	+	+	+	-
[38]	+	-	+	?	?	-	-	-	+	+	?	-
[39]	+	-	+	+	+	?	-	-	+	+	+	-
[50]	+	-	+	-	+	-	-	-	+	+	+	-
[37]	+	-	+	+	+	?	-	-	+	+	+	-
[98]	+	-	+	?	?	?	-	-	+	+	+	-
[101]	+	-	+	+	+	?	-	-	+	+	+	-
[103]	+	-	+	+	+	?	-	-	+	+	+	-
[74]	+	-	+	?	?	-	-	-	+	+	+	-
[75]	+	-	?	?	?	+	-	+	+	+	+	-
[71]	+	-	-	+	+	+	-	+	+	+	+	-
[72]	+	-	+	+	+	?	-	-	+	+	+	-

[76]	+	-	?	?	?	?	-	+	+	+	?	-
[73]	+	+	+	?	+	?	-	-	+	+	-	-

Green dot = clearly reported. The item is fully and explicitly described, allowing readers to assess bias and applicability without ambiguity. This aligns with STARD's goal of transparency and completeness. Yellow dot = unclear reporting: the item is mentioned but lacks sufficient detail. STARD emphasizes that vague or partial reporting impairs reproducibility and critical appraisal. Red dot = not reported: the item is omitted entirely.
